# Beyond seizure control: Identifying deficits in cognitive networks in absence epilepsy

**DOI:** 10.1126/sciadv.aed3642

**Published:** 2026-05-13

**Authors:** Gil Vantomme, Gabrielle Devienne, Jacob M. Hull, John R. Huguenard

**Affiliations:** Department of Neurology and Neurological Sciences, Stanford University, Stanford, CA, USA.

## Abstract

Cognitive impairments are common in absence epilepsy, yet the neural basis of these deficits remains largely unknown. Communication between the thalamic reuniens nucleus and the prefrontal cortex is critical for flexible behavior. Here, we identify a disruption of this pathway in mice with absence epilepsy. This dysfunction leads to impaired cognitive flexibility and altered cortical inhibition, linking seizure-related thalamic activity to prefrontal network imbalance. Notably, targeted stimulation of the thalamic-prefrontal pathway alleviates both seizure occurrence and cognitive deficits. These findings reveal a shared circuit mechanism underlying epileptic and cognitive symptoms, pointing to new strategies for intervention.

## Teaser

Disrupted thalamic-prefrontal communication ties seizures to cognitive deficits in absence epilepsy.

## INTRODUCTION

Typical absence seizures are characterized by sudden, brief lapses of consciousness accompanied by behavioral arrest and stereotypical spike-and-wave discharges (SWDs) (*1*, *2*). Beyond the immediate impact of seizures, affected individuals frequently experience neuropsychiatric comorbidities, including deficits in attention, memory, and cognitive flexibility, as well as mood impairments [for review: (*3*, *4*)]. A substantial fraction of patients remains pharmaco-resistant, and comorbidities often persist even when seizures are controlled (*5*–*7*). These challenges highlight the need to elucidate the mechanisms underlying both seizure activity and associated cognitive deficits, with the goal of developing treatments that address the full spectrum of clinical burden.

The thalamocortical network is a critical driver of typical absence seizures (*8*–*10*), yet its impact is not uniformly distributed across brain regions. SWDs generalize rapidly across hemispheres and cortices, with initial foci in somatosensory and frontal/motor areas reported across species (*11*–*14*). Limbic structures such as the hippocampus show transient and focal increases in BOLD signals during SWD (*15*), increase cerebral glucose utilization rates (*16*), and increase synchronicity without full paroxysmal discharge (*17*) in typical absence epilepsy, suggesting that they are relatively spared compared to sensory motor areas. This is in contrast with atypical absence epilepsy, where midline thalamus and hippocampus are highly involved in seizure generation [for review: (*18*)].

This raises the possibility that circuit-specific dysfunctions outside the canonical seizure network contribute to the cognitive comorbidities in typical absence epilepsy. Here, we focused on the reuniens nucleus of the thalamus (Re). The Re is a midline thalamic nucleus that serves as a critical hub for prefrontal-hippocampal communication and state-dependent thalamocortical synchronization (*19*). These functions are directly relevant to absence epilepsy, which is characterized not only by pathological thalamocortical oscillations but also by impairments in cognitive control and executive function (*20*). The medial prefrontal cortex (mPFC) plays a central role in these cognitive processes, making Re projections to mPFC a candidate circuit through which seizure-related network dysfunction could contribute to cognitive comorbidities. In addition to its functional relevance, the Re has emerged as a promising neuromodulatory target in neurological disease. Manipulation of Re activity has been explored in models of Alzheimer’s disease (*21*) and genetic epilepsy, including BK-D434G channelopathy, where midline thalamic stimulation reduced seizure activity (*22*). Together, these observations motivated our focus on the Re-mPFC circuit as a convergence point for thalamocortical network dysfunction, cognitive impairment, and therapeutic intervention in Scn8a^+/−^ mice.

We investigated the Scn8a^+/−^ mouse model of absence epilepsy, which carries a heterozygous loss-of-function mutation in the voltage-gated sodium channel NaV1.6. This mutation leads to hypersynchronous activity in thalamocortical networks and the emergence of cortical SWDs (*23*). The Scn8a^+/−^ model is a well-established model of absence epilepsy that exhibits frequent seizures and attentional deficits resembling cognitive comorbidities observed in patients (*24*). Using a combination of in vitro and in vivo electrophysiological recordings and optogenetic manipulation, we examined circuit abnormalities between the Re and the mPFC, their impact on SWDs, and cognitive flexibility.

We show that Scn8a^+/−^ mice exhibit impaired cognitive flexibility, recapitulating a key comorbidity observed in patients. Despite minimal engagement of mPFC units in SWDs and the absence of seizure induction by Re stimulation, the synaptic recruitment of the mPFC by the Re is disrupted. Our data suggest that this deficit arises from reduced feedforward inhibition, in part due to the hypoexcitability of mPFC layer 1 (L1) interneurons. Notably, optogenetic stimulation of Re at 20 Hz reduced seizure incidence in vivo and restored performance during reversal learning.

Together, these findings reveal that the Re-mPFC circuit is functionally altered in absence epilepsy and may underlie cognitive comorbidities. They further demonstrate that targeted stimulation of Re is both safe and effective in suppressing seizures, identifying this pathway as a promising candidate for circuit-based therapeutic interventions in patients with refractory epilepsy.

## RESULTS

### SWDs are largely absent in the mPFC of Scn8a^+/−^ mice

**Fig. 1.**
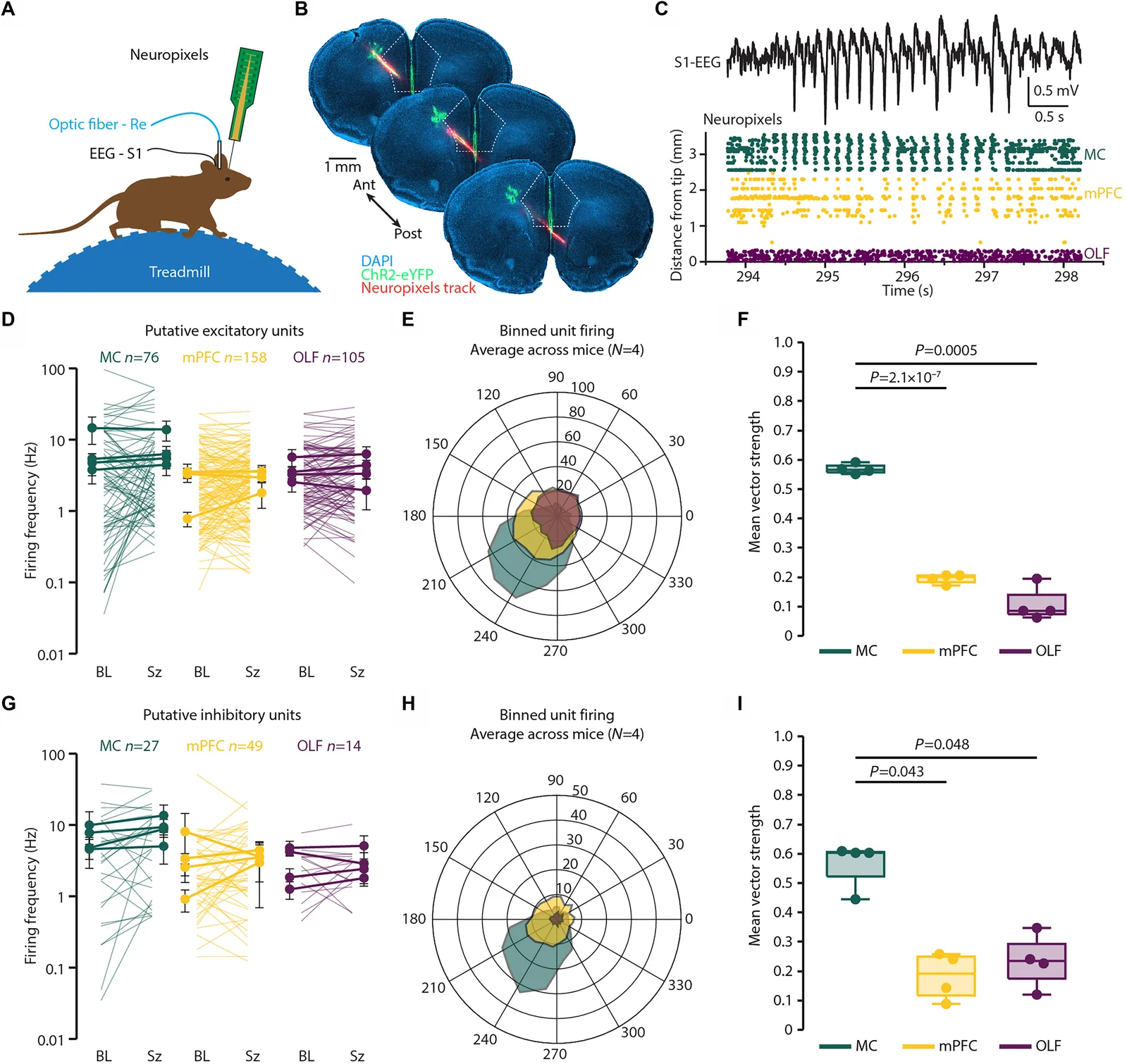
SWDs are largely absent in the mPFC of Scn8a^+/−^ mice. (**A**) Schematic of the recording setup: acute Neuropixels probe in the frontal cortex, EEG in primary somatosensory cortex (S1), and optic fiber targeting the Re. Mice were awake and head restrained on a cylindrical treadmill. (**B**) Epifluorescence images of mPFC coronal slices showing ChR2-eYFP–labeled Re afferents (green), Neuropixels probe tracks (red), and DAPI-stained nuclei (blue). (**C**) Example recording from a prototypical Scn8a^+/−^ mouse. SWD is recorded with EEG lead (top) along with corresponding unit activity from the Neuropixels probe in the MC, mPFC, and OLFs (bottom). (**D**) Firing frequency of putative excitatory units across brain areas during the interictal period (BL) and during seizures (Sz). Solid lines represent averages for each mouse (*N* = 4). Transparent lines show individual units. (**E**) Average phase plot of unit firing in the MC (green), mPFC (yellow), and OLFs (magenta) during SWDs. (**F**) Mean vector strength for analysis in (E). (**G**) Same as (D) for putative inhibitory units. (**H**) Same as (E) for putative inhibitory units. (**I**) Same as (F) putative inhibitory units.

The mPFC has been reported as a region involved in SWD during typical absence seizures, either as a propagation site (*25*) or even as a focal onset zone in a GHB (γ-hydroxybutyrate) mouse model (*14*). To examine this in Scn8a^+/−^ mice, we analyzed acute Neuropixels recording from single units in the motor cortex (MC), mPFC, and olfactory bulbs (OLFs) during seizures in a cohort of five Scn8a^+/−^ and three wild-type littermates. Four of the five Scn8a^+/−^ mice showed at least one seizure during the acute recording (Fig. 1, A to C; *N* = 4 mice). We then plotted unit firing rates relative to the phase of the SWDs (Fig. 1D). The unit spike width was used to separate putative excitatory and inhibitory cells. Units with a spike width equal to or below 0.37 ms were considered as putative inhibitory cells. Putative excitatory units in the MC (*n* = 76), mPFC (*n* = 158), and OLFs (*n* = 105) did not change their average firing rate between interictal (MC: 7.1 ± 2.5; mPFC: 2.8 ± 0.5; OLF: 3.7 ± 0.9) and SWD (MC: 7.5 ± 2.2; mPFC: 3.0 ± 0.6; OLF: 4.0 ± 1.0) periods (Fig. 1D; paired Student’s *t* tests, *N* = 4, MC: *P* = 0.39; mPFC: *P* = 0.53; OLF: *P* = 0.53). However, plotting the unit firing rate against the phase of the SWDs revealed strong phase locking to the spike component in the MC, with a mean vector strength of 0.57 ± 0.01. By contrast, units in mPFC and OLFs showed little phase locking, with mean vector strengths of 0.19 ± 0.01 and 0.11 ± 0.03, respectively [one-way repeated-measures analysis of variance (ANOVA): *F*_2,6_ = 207.7, *P* = 6.0 × 10^−8^; post hoc paired Student’s *t* tests with Hochberg correction: MC-mPFC, *P* = 2.1 × 10^−7^; MC-OLF, *P* = 0.0005; mPFC-OLF, *P* = 0.056) (Fig. 1, E and F). Putative inhibitory units in the MC (*n* = 27), mPFC (*n* = 49), and OLFs (*n* = 14) also showed no change in their average firing rate between interictal (MC: 6.5 ± 2.7; mPFC: 3.6 ± 1.9; OLF: 2.9 ± 0.6) and SWD (MC: 8.8 ± 2.7; mPFC: 3.5 ± 1.5; OLF: 2.9 ± 1.1) periods (Fig. 1G; paired Student’s *t* tests, *N* = 4; MC: *P* = 0.07; mPFC: *P* = 0.95; OLF: *P* = 0.97). These putative inhibitory units also showed strong phase locking to the spike of the SWD in the MC (0.57 ± 0.04) but not mPFC (0.18 ± 0.04) and OLFs (0.38 ± 0.05) (Fig. 1, H and I; Kruskal-Wallis test, *P* = 0.024; post hoc Dunn tests with Hochberg correction: MC-mPFC, *P* = 0.043; MC-OLF, *P* = 0.048; mPFC-OLF, *P* = 0.84). This weaker phase locking of mPFC units indicates that SWDs are not effectively recruiting or synchronizing neuronal ensembles in this region during ictal events in Scn8a^+/−^ mice, contrasting with regions such as the MC, where strong phase coupling is observed.

We compared interictal firing rates of putative excitatory and inhibitory units across brain areas between Scn8a^+/−^ mice (*N* = 5) and wild-type littermates (Scn8a^+/+^, *N* = 3; Table 1 and fig. S1, A and B). Interictal firing rates were similar between genotypes across all brain areas and unit types, except for putative excitatory units in the mPFC. In this region, Scn8a^+/−^ mice exhibited a lower average interictal firing rate compared to wild-type littermates (Scn8a^+/+^: 5.2 ± 0.7 Hz, *N* = 3; Scn8a^+/−^: 2.8 ± 0.6 Hz, *N* = 5; *F*_1,6_ = 6.8, *P* = 0.04).

**Table 1.** Baseline firing rate of putative excitatory and inhibitory units in Scn8a^+/−^ and wild-type littermates. Units were recorded acutely with a Neuropixels probe in awake, head-restrained Scn8a^+/−^ mice and wild-type littermates on a cylindrical treadmill. Frequencies are expressed as the means ± SEM. na, not applicable.

Baseline/interictal unit firing		Putative excitatory					
		MC		mPFC		OLF	
Mouse	Geno	Unit (#)	Avg freq (Hz)	Unit (#)	Avg freq (Hz)	Unit (#)	Avg freq (Hz)
M0586	Scn8a^+/+^	2	1.1 ± 0.4	57	3.5 ± 0.3	0	na
M0694	Scn8a^+/+^	70	5.8 ± 1.3	46	5.9 ± 1.4	20	2.0 ± 0.5
M0702	Scn8a^+/+^	26	4.6 ± 1.0	12	6.3 ± 2.4	4	3.1 ± 1.1
M0700	Scn8a^+/−^	34	5.4 ± 1.0	26	3.5 ± 1.0	39	3.5 ± 0.7
M0703	Scn8a^+/−^	16	14.6 ± 6.1	5	0.8 ± 0.2	15	5.7 ± 1.6
M3018	Scn8a^+/−^	20	4.7 ± 1.6	69	3.3 ± 0.4	11	2.5 ± 0.7
M3020	Scn8a^+/−^	6	3.8 ± 1.4	58	3.5 ± 0.5	40	3.2 ± 0.5
M3023	Scn8a^+/−^	12	5.2 ± 2.2	52	2.9 ± 0.8	5	5.5 ± 2.4
Baseline/interictal unit firing		Putative inhibitory					
		MC		mPFC		OLF	
Mouse	**Geno**	**Unit (#)**	**Avg freq (Hz)**	**Unit (#)**	**Avg freq (Hz)**	**Unit (#)**	**Avg freq (Hz)**
M0586	Scn8a^+/+^	0	na	4	2.8 ± 1.1	0	na
M0694	Scn8a^+/+^	8	5.2 ± 0.8	5	2.9 ± 1.3	1	3.6
M0702	Scn8a^+/+^	4	3.1 ± 2.7	6	2.8 ± 1.2	0	na
M0700	Scn8a^+/−^	7	9.5 ± 5.1	8	7.7 ± 6.2	5	1.2 ± 0.3
M0703	Scn8a^+/−^	7	7.5 ± 2.4	3	0.9 ± 0.3	2	4.0 ± 0.4
M3018	Scn8a^+/−^	11	4.4 ± 2.0	20	2.4 ± 0.6	3	1.8 ± 0.5
M3020	Scn8a^+/−^	2	4.6 ± 1.4	18	3.3 ± 0.5	4	4.6 ± 1.1
M3023	Scn8a^+/−^	1	0.3	8	3.3 ± 1.1	3	2.4 ± 2.1

To assess whether the Re recruitment of mPFC units was altered in Scn8a^+/−^ mice, we optogenetically stimulated the Re (fig. S1C) while recording evoked responses in the mPFC in the same mice (fig. S1D and Table 2). Consistent with a previous report (*26*), Re activation elicited both single-spike and burst firing in mPFC units (fig. S1, D and E), with similar proportions across genotypes and putative cell types (fig. S1E; excitatory: chi-square test, *P* = 0.5; inhibitory: chi-square test, *P* = 0.08). Because the number of responding putative inhibitory units was insufficient for further analysis, subsequent analyses focused on excitatory units. In both genotypes, Re stimulation increased the average firing rate of putative excitatory units (fig. S1F), which showed comparable response latencies and bursting properties (fig. S1, G and H).

**Table 2.** Firing properties of putative excitatory and inhibitory units in mPFC upon optogenetic activation of Re. Units were recorded acutely with a Neuropixels probe in awake, head-restrained Scn8a^+/−^ mice and wild-type littermates on a cylindrical treadmill. The Re was stimulated through an optic fiber positioned above the Re itself. Frequencies are expressed as the means ± SEM.

Putative excitatory units								
Mouse	Geno	Unit (#)	Pre stim freq (Hz)	Latency to spike (ms)	Post stim freq (Hz)	Bursting units (#)	Spike in burst (#)	Avg inst freq (Hz)
M0586	Scn8a^+/+^	6	5.7 ± 1.0	12.7 ± 3.9	30 ± 24.4	3	2.8 ± 0.8	138.7 ± 46.9
M0694	Scn8a^+/+^	5	9.0 ± 3.6	16.1 ± 1.5	25.3 ± 8.6	4	2.1 ± 0.0	111.8 ± 16.9
M0702	Scn8a^+/+^	8	10.5 ± 4.9	7.4 ± 1.7	25.5 ± 12.0	6	2.2 ± 0.2	172.7 ± 26.9
M0700	Scn8a^+/−^	2	6.5 ± 0.5	15.7 ± 1.1	13.7 ± 8.8	1	2.5	96.4
M0703	Scn8a^+/−^	0	na	na	na	0	na	na
M3018	Scn8a^+/−^	19	5.1 ± 1.0	12.8 ± 1.1	8.3 ± 3.0	8	2.2 ± 0.1	218.6 ± 50.5
M3020	Scn8a^+/−^	8	5.6 ± 2.1	13.6 ± 2.7	13.0 ± 8.4	3	2.2 ± 0.2	157.4 ± 36.1
M3023	Scn8a^+/−^	23	7.2 ± 2.3	12.9 ± 1.0	31.3 ± 7.8	19	2.3 ± 0.1	184.8 ± 35.2
Putative inhibitory units								
Mouse	**Geno**	**Unit (#)**	**Pre stim freq (Hz)**	**Latency to spike (ms)**	**Post stim freq (Hz)**	**Bursting units (#)**	**Spike in burst (#)**	**Avg inst freq (Hz)**
M0586	Scn8a^+/+^	0	na	na	na	0	na	na
M0694	Scn8a^+/+^	0	na	na	na	0	na	na
M0702	Scn8a^+/+^	1	7.3	14.4	9.4	0	na	na
M0700	Scn8a^+/−^	0	na	na	na	0	na	na
M0703	Scn8a^+/−^	1	0.7	20.5	0.7	0	na	na
M3018	Scn8a^+/−^	4	1.8 ± 1.0	10.1 ± 2.1	25.9 ± 18.4	2	2.3 ± 0.9	219.6 ± 97.9
M3020	Scn8a^+/−^	5	5.1 ± 1.4	9.3 ± 2.7	16.3 ± 9.3	2	2.2 ± 0.2	95.1 ± 1.9
M3023	Scn8a^+/−^	3	7.8 ± 2.1	17.0 ± 2.3	18.7 ± 8.5	2	2.4 ± 0.2	124.0 ± 12.4

### Re recruitment of the mPFC is altered in the Scn8a^+/−^ mouse model of absence seizures

**Fig. 2.**
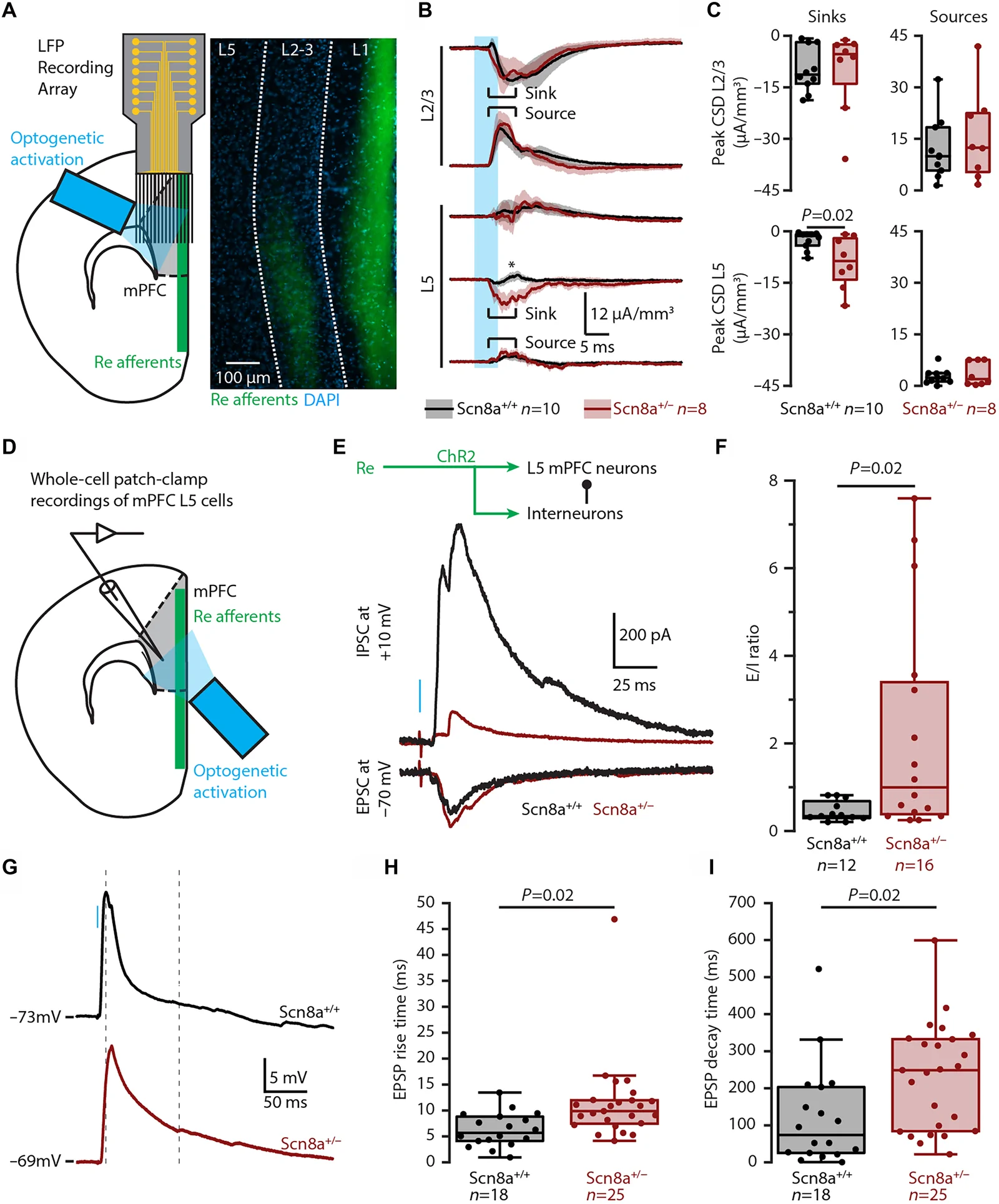
Re recruitment of the mPFC is altered in the Scn8a^+/−^ mice. (**A**) Left: Recording configuration for LFPs. A 16-channel silicon probe was positioned across cortical layers of the mPFC in acute coronal slices from Scn8a^+/−^ and wild-type littermates expressing ChR2 in the Re. Right: Epifluorescence micrograph showing the eYFP/ChR2 fibers (green) from the Re projecting to the mPFC. (**B**) Example CSD profiles evoked by optogenetic Re activation. (**C**) Peak CSD sinks and sources in L2/3 (top) and L5 (bottom) of the mPFC. (**D**) Recording configuration for whole-cell patch clamp: L5 pyramidal neurons in mPFC were recorded in acute slices with ChR2-expressing Re afferents. (**E**) Example traces of monosynaptic EPSCs (−70 mV) and polysynaptic IPSCs (+10 mV) evoked by Re optogenetic stimulation. (**F**) E/I ratio calculated as EPSC amplitude (−70 mV) divided by IPSC amplitude (+10 mV). (**G**) Example current-clamp traces showing subthreshold EPSPs evoked by Re stimulation. (**H** and **I**) Quantification of EPSP rise time (H) and decay time (I).

Although units in the mPFC show little phase locking to the SWDs, the lower average firing rate of putative excitatory units in our Scn8a^+/−^ model and the presence of cognitive deficits in patients with absence epilepsy strongly suggest dysfunction in associated circuits (*3*, *24*). To test whether the Re-mPFC pathway relevant for cognition is affected in Scn8a^+/−^ mice, we recorded local field potentials (LFPs) in the mPFC using a 16-shank linear electrode array spanning cortical layers (Fig. 2A). Slices were obtained from mice expressing channelrhodopsin-2 (ChR2) in Re axons after adeno-associated virus serotype 1 (AAV1)–CaMKIIα (calcium- and calmodulin-dependent protein kinase IIα)–ChR2–enhanced yellow fluorescent protein (eYFP) injection into the Re. Blue light activation of Re afferents evoked LFPs across mPFC layers, from which we derived current source density (CSD) maps (Fig. 2B) (*24*, *27*).

In the CSD signals, pairs of sinks and sources were observed following optogenetic stimulation of Re afferents, attributed to cations flowing from the extracellular space into the cells during synaptic excitation and flowing back out of cells at a distance, respectively. Sources and sinks were prominent within the first 10 ms after the light onset in L2/3 and L5 (Fig. 2C). L2/3 peak CSD sinks (Scn8a^+/+^: −10 ± 2 μA/mm^3^, *n* = 10; Scn8a^+/−^: −10 ± 4 μA/mm^3^, *n* = 8; *F*_1,16_ = 0.001, *P* = 0.98) and sources (Scn8a^+/+^: 12 ± 3 μA/mm^3^, *n* = 10; Scn8a^+/−^: −16 ± 5 μA/mm^3^, *n* = 8; *F*_1,16_ = 0.43, *P* = 0.52) did not differ between genotypes. By contrast, Scna8^+/−^ showed a significantly larger sink in L5 (Scn8a^+/+^: −3 ± 1 μA/mm^3^, *n* = 10; Scn8a^+/−^: −9 ± 3 μA/mm^3^, *n* = 8; *F*_1,16_ = 6.31, *P* = 0.02), indicating altered recruitment of the deeper, output layer of mPFC.

To determine the specific circuit alterations underlying the global synaptic source/sink differences, we performed whole-cell patch-clamp recordings from the major output neurons of the mPFC, L5 pyramidal neurons (Fig. 2D). Optogenetic stimulation of Re axons evoked excitatory postsynaptic currents (EPSCs) in L5 cells at −70 mV (near the Cl^−^ reversal potential) and inhibitory postsynaptic currents (IPSCs) at +10 mV [near the AMPAR (α-amino-3-hydroxy-5-methyl-4-isoxazolepropionic acid receptor)/NMDAR (*N*-methyl-d-aspartate receptor) reversal potential] (Fig. 2E). EPSC latencies were short (Scn8a^+/+^: 4.0 ± 0.3 ms, *n* = 12; Scn8a^+/−^: 4.7 ± 0.5 ms, *n* = 16; *F*_1,26_ = 1.48, *P* = 0.2), consistent with monosynaptic input, whereas IPSC latencies were longer (Scn8a^+/+^: 8.0 ± 0.5 ms, *n* = 12; Scn8a^+/−^: 10.2 ± 0.9 ms, *n* = 16; *F*_1,26_ = 3.92, *P* = 0.06), consistent with polysynaptic recruitment (fig. S2).

Peak EPSC amplitudes were similar across genotypes (Scn8a^+/+^: −331 ± 80 pA; Scn8a^+/−^: −331 ± 68 pA; *F*_1,26_ = 0.000, *P* = 1.0). However, IPSC amplitudes were reduced in Scn8a^+/−^ (782 ± 137 pA) compared to their wild-type littermates (422 ± 102 pA; *F*_1,26_ = 4.64, *P* = 0.041). This resulted in a marked shift in the excitatory/inhibitory (E/I) ratio: Scn8a^+/+^ mice showed the classical strong feedforward inhibition (E/I = 0.46 ± 0.07), whereas Scn8a^+/−^ cells showed a significantly elevated E/I ratio (2.22 ± 0.62; *F*_1,26_ = 5.95, *P* = 0.02), with about half of Scn8a^+/−^ neurons showing little to no feedforward inhibition (Fig. 2F). This synaptic imbalance toward excitation was reflected in prolonged excitatory postsynaptic potentials (EPSPs) recorded in current clamp at resting membrane potential (Fig. 2, G to I). Repetitive 10-Hz stimulation of Re afferents produced short-term depression of both EPSCs and IPSCs in both genotypes (fig. S3).

**Fig. 3.**
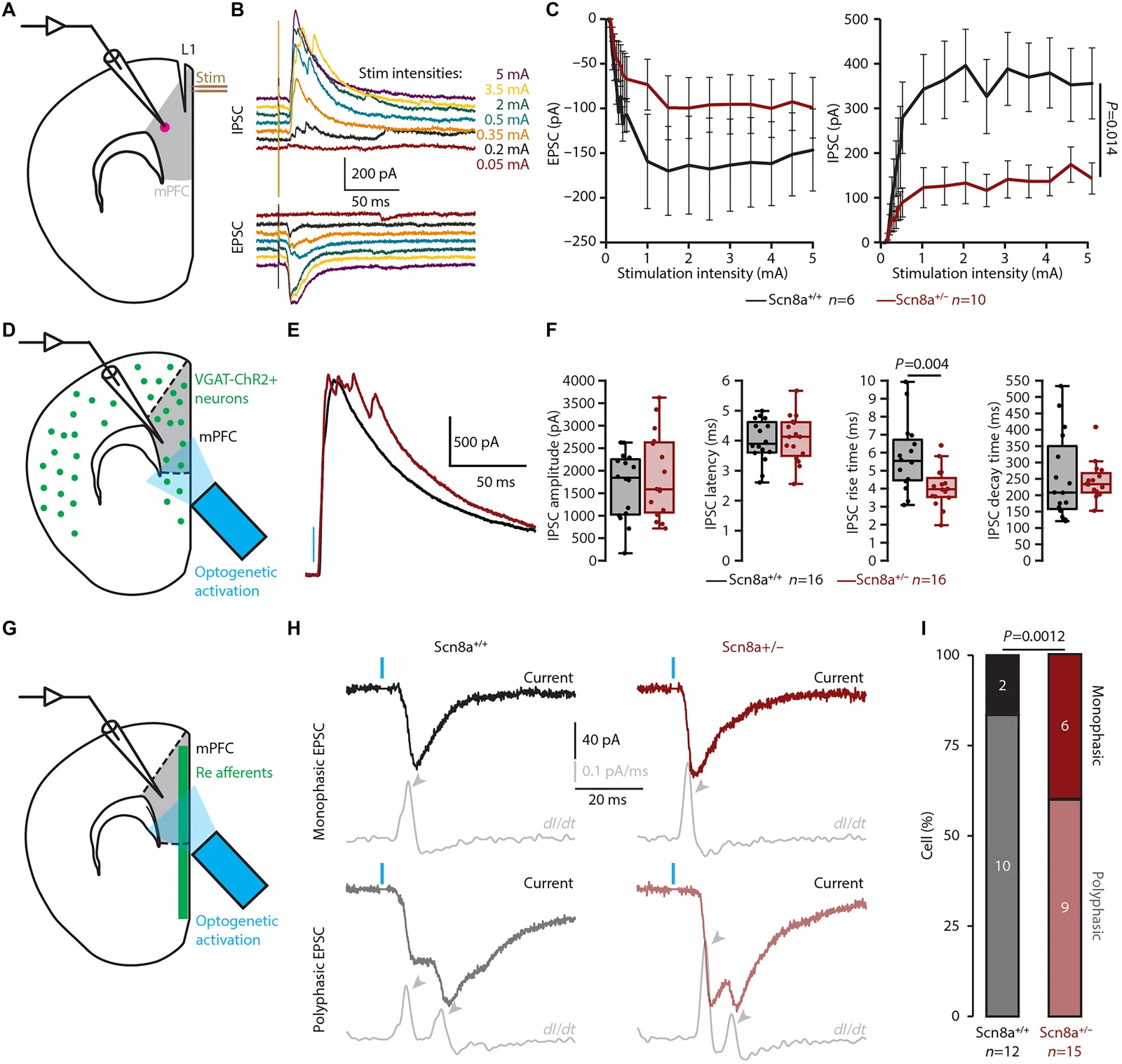
Re recruitment of the mPFC is altered in the Scn8a^+/−^ mice. (**A**) Whole-cell patch recordings from L5 pyramidal neurons in mPFC slices with a partially disconnected L1 flap. A bipolar electrode delivered electrical stimulation in the L1 flap. (**B**) Example EPSCs (−70 mV) and IPSCs (+10 mV) evoked by increasing stimulation intensities. (**C**) Quantification of EPSC and IPSC amplitudes. (**D**) Whole-cell patch recordings from L5 pyramidal neurons in acute slices from Scn8a^med^-VGAT-ChR2 mice. (**E**) Example IPSCs evoked by optogenetic activation of local interneurons. (**F**) Quantification of IPSC properties. (**G**) Whole-cell patch recordings from L5 pyramidal neurons in slices expressing ChR2 in Re afferents. (**H**) Example monophasic and polyphasic EPSCs evoked by optogenetic activation of Re afferents. (**I**) Proportion of L5 neurons responding to Re afferent stimulation with monophasic versus polyphasic EPSCs.

Because absolute amplitudes of optogenetically evoked currents can vary with ChR2 expression levels, we used a semi-isolated L1 flap preparation to allow more controlled and comparable activation of L1 inputs. In the mPFC, L1, where Re axons are most heavily concentrated (*28*), was partially disconnected by a cut, preserving local L1 circuitry while limiting polysynaptic spread to adjacent columns (*29*). A bipolar electrical stimulation electrode was positioned in the semi-isolated L1 flap, while recordings were made from L5 pyramidal neurons in an adjacent intact column (Fig. 3A). This preparation enables precise control of stimulation intensity and spatial restriction of L1 activation (*29*) while maintaining the natural dendritic and interneuron-mediated pathways through which L1 inputs influence deep-layer pyramidal neurons. As a result, this preparation allows us to assess how effectively L1-driven circuits rather than variable levels of ChR2 expression recruit inhibitory and excitatory responses in the mPFC network (Fig. 3B). In this configuration, Scn8a^+/−^ mice showed similar EPSC amplitudes compared to wild-type littermates (Scn8a^+/+^: −147 ± 46 pA, *n* = 6; Scn8a^+/−^: −99 ± 34 pA, *n* = 10, *F*_1,14_ = 0.71, *P* = 0.41). However, IPSCs in Scn8a^+/−^ mice plateaued rapidly with increasing stimulation intensity and were significantly smaller than in controls (Scn8a^+/+^: 356 ± 79 pA, *n* = 6; Scn8a^+/−^: 143 ± 35 pA, *n* = 10, *F*1,14 = 7.94, *P* = 0.014) (Fig. 3C). These results show that electrical stimulation of mPFC L1 evokes similar monosynaptic EPSC amplitudes in L5 pyramidal neurons across genotypes but smaller polysynaptic IPSC amplitudes in Scn8a^+/−^ mice, consistent with the findings from optogenetic stimulation of Re afferents (Fig. 2 and fig. S2). This suggests an altered recruitment of inhibitory circuits in the mPFC.

To test whether this deficit reflected impaired interneuron output, we directly activated local interneurons by optogenetic stimulation in Scn8a^med^-VGAT-ChR2 mice (Fig. 3D). IPSC properties were similar across genotypes (Fig. 3, E and F), apart from a slight decrease in rise time in Scn8a^+/−^, suggesting that the E/I imbalance in Scn8a^+/−^ arises from reduced polysynaptic recruitment rather than interneuron output deficits.

A particular feature of the Re-mPFC circuit is its propensity to recruit feedforward excitation in addition to the classical feedforward inhibition, as indicated by polyphasic EPSCs (*26*). Scn8a^+/−^ mice exhibited a lower proportion of pyramidal cells with polyphasic EPSCs (9 of 15 cells) compared with wild-type littermates (10 of 12 cells; chi-square test, *P* = 0.001) (Fig. 3, G to I). Notably, the proportion of cells (83%) with polysynaptic Re-driven excitation in these wild-type C3Fe.Cg mice is higher than the proportion previously reported in C57BL6/J mice (63%) (*26*), suggesting that the prevalence of these polysynaptic events may vary with the genetic background. These reductions in both polysynaptic feedforward excitation and inhibition upon stimulation of Re afferents, together with relatively preserved monosynaptic excitation, suggest increased failures in action potential (AP) generation or propagation in Scn8a^+/−^ mice, consistent with the NaV1.6 loss-of-function mutation.

### L1 mPFC interneurons are hypoexcitable in Scn8a^+/−^ mice

Beyond the synaptic deficits we observed above, changes in cellular excitability may also contribute to the L5 deficits in the mPFC of Scn8a^+/−^ mice. Previous work showed that the activity of mPFC parvalbumin interneurons was affected and directly linked to attentional deficits (*24*). L5 pyramidal cells themselves showed little to no changes in excitability (fig. S4). The L1 of the cortex contains a sparse but distinct population of GABAergic interneurons situated at the cortical surface (*30*). These interneurons regulate integration of top-down signals from cortical and subcortical areas, making them critical gatekeepers of input and modulators of deeper-layer activity (*31*). Their ability to filter incoming signals positions them as potential first responders in containing abnormal activity that may precede or trigger seizures. L1 is also a major termination zone for Re axons, which target the apical dendrites of deep-layer pyramidal neurons. Positioned near these apical dendrites, L1 interneurons are therefore well suited to regulate the integration of Re-driven excitatory input and to modulate the impact of thalamocortical signals on deeper cortical layers.

**Fig. 4.**
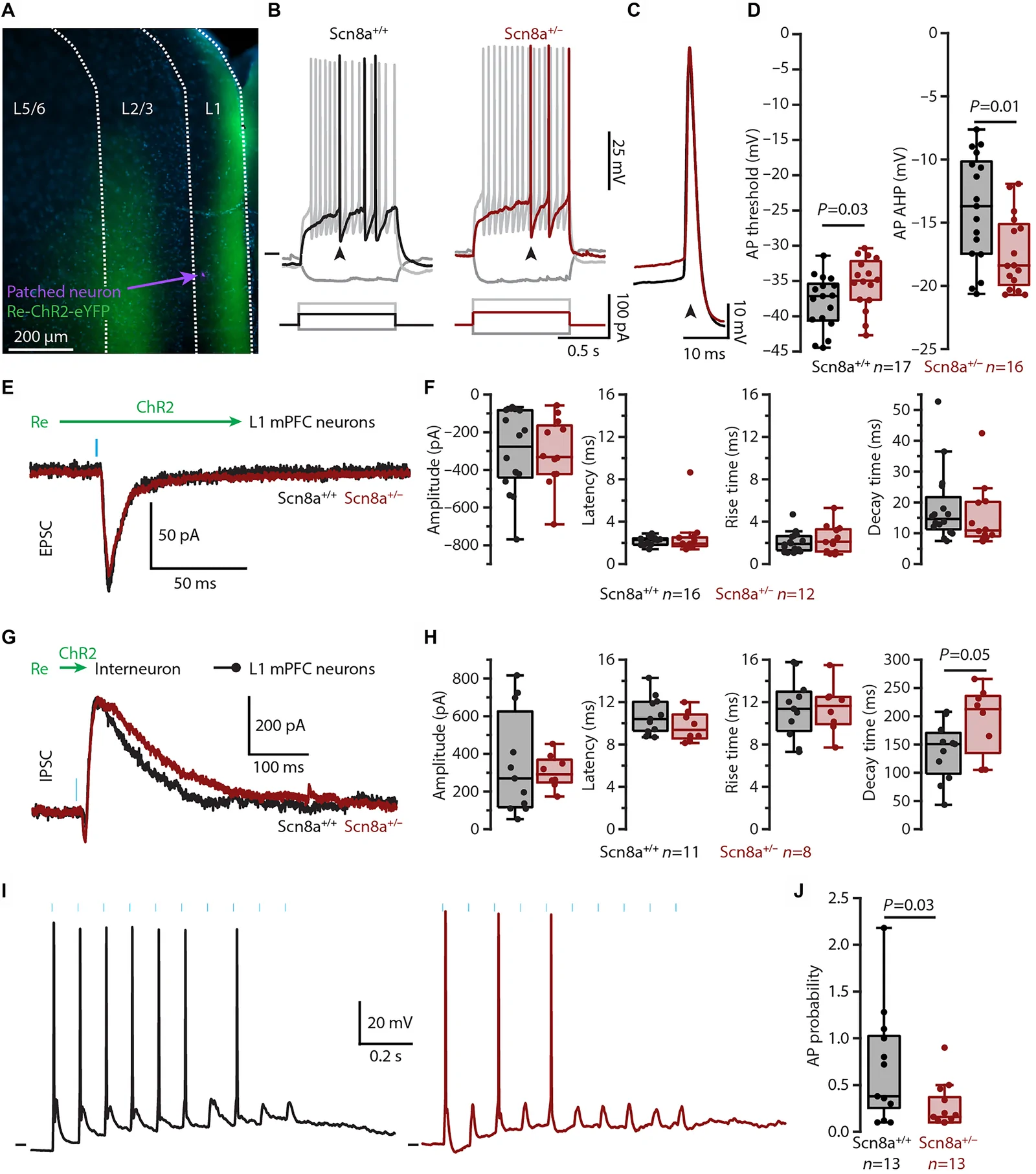
L1 mPFC interneurons are hypoexcitable in Scn8a^+/−^ mice. (**A**) Epifluorescence micrograph of an mPFC slice showing recorded L1 interneuron (purple), Re afferents expressing ChR2-eYFP (green), and DAPI-stained nuclei (blue). (**B**) Firing patterns of L1 interneurons. (**C**) Representative action potentials. (**D**) Quantification of action potential (AP) threshold and afterhyperpolarization. (**E**) EPSCs recorded in L1 interneurons. (**F**) Quantification of EPSC properties. (**G**) IPSCs recorded in L1 interneurons. (**H**) Quantification of IPSC properties. (**I**) Action potential discharge from resting membrane potential during 10-Hz train stimulation of Re afferents. (**J**) Quantification of action potential discharge probability during each stimulus within a 10-Hz train of stimuli.

Whole-cell recordings from L1 interneurons (Fig. 4A) revealed a late-firing pattern in most cases, consistent with a prior report on neurogliaform cells (Fig. 4B) (*31*). Scn8a^+/−^ interneurons showed a higher action potential threshold at the rheobase (−35.5 ± 0.9 mV, *n* = 16) compared to wild-type littermates (−38.4 ± 0.9 mV, *n* = 17; *F*_1,31_ = 5.21, *P* = 0.03) and lower action potential afterhyperpolarization (Scn8a^+/−^: −17.4 ± 0.7 mV, *n* = 16; Scn8a^+/+^: −13.9 ± 1.0 mV, *n* = 17; *F*_1,31_ = 7.15, *P* = 0.01) (Fig. 4, C and D). The depolarized spike threshold is consistent with a direct effect of NaV1.6 loss of function, whereas the altered afterhyperpolarization may reflect additional cell type–specific intrinsic adaptations.

Optogenetic activation of Re afferents evoked monosynaptic EPSCs in L1 interneurons with comparable latency and kinetics across genotypes (Fig. 4, E and F). Feedforward polysynaptic IPSCs were also broadly similar, although Scn8a^+/−^ mice showed a modest increase in decay time (Scn8a^+/−^: 192 ± 22 ms, *n* = 8; Scn8a^+/+^: 138 ± 16 ms, *n* = 11; *F*_1,17_ = 4.45, *P* = 0.05) (Fig. 4, G and H).

When Re afferents were repeatedly stimulated at 10 Hz, L1 interneurons fired action potentials and subthreshold EPSPs from their resting membrane potential (Fig. 4I). We quantified the number of spikes elicited during a train of 10 stimuli at 10 Hz, considering only recordings in which the first stimulus triggered an action potential. Consistent with their elevated threshold, Scn8a^+/−^ interneurons fired significantly fewer action potentials than controls (Scn8a^+/−^: 0.26 ± 0.07, *n* = 13; Scn8a^+/+^: 0.68 ± 0.17, *n* = 13; *F*_1,24_ = 5.38, *P* = 0.03) (Fig. 4J). Together, these findings demonstrate that L1 interneurons in Scn8a^+/−^ mice are hypoexcitable, reducing their ability to respond to Re input and thereby weakening inhibitory control at the cortical entry point.

### Re stimulation reduces seizure incidence in Scn8a^+/−^ mice

**Fig. 5.**
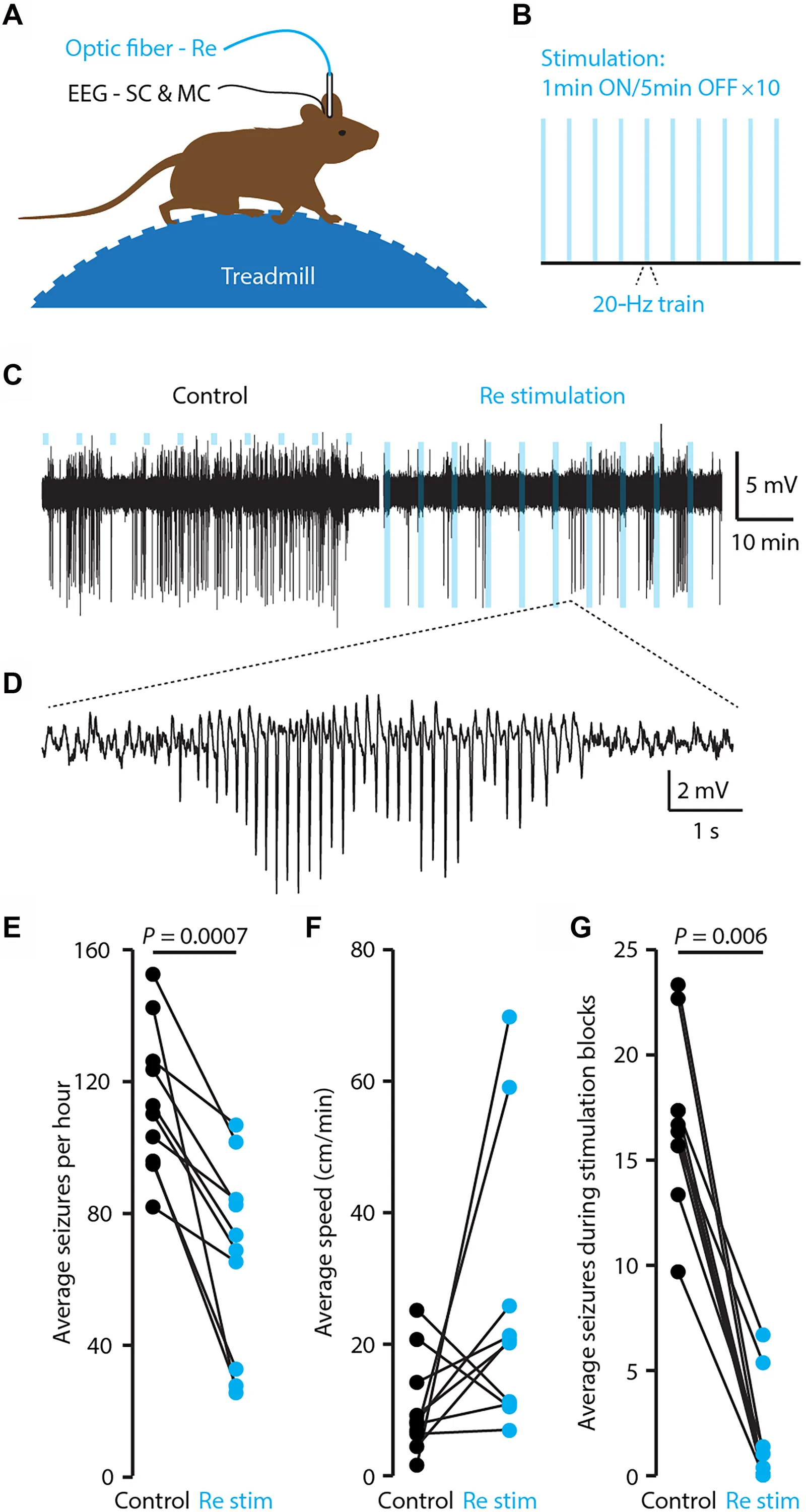
Re stimulation reduces seizure incidence in Scn8a^+/−^ mice. (**A**) Recording configuration: EEG electrodes in the somatosensory cortex (SC) and MC, with optic fiber targeting the Re. Mice were awake and head restrained on a cylindrical treadmill. (**B**) Stimulation protocol: 10 repetitions of a 1-min, 20-Hz train followed by 5 min without stimulation (total duration, 1 hour). (**C**) EEG traces from a 1-hour recording. Blue bars mark periods of light delivery in the recording cage (left, control stimulation) or on the Re (right). (**D**) Expanded EEG traces from (C) showing a typical SWD. (**E**) Average number of seizures during 1-hour-long recordings in control and Re stimulation conditions. (**F**) Average speed on the cylindrical treadmill during 1-hour-long recordings in control and Re stimulation conditions. (**G**) Same as (E) restricted to the 10 blocks of stimulation.

Mice implanted chronically for electroencephalography (EEG) recordings were trained to rest while head-fixed on a cylindrical treadmill (Fig. 5A). Optogenetic stimulation was 1 min of 20-Hz train followed by 5 min without stimulation, alternating for 1 hour (Fig. 5B). Blue light was delivered to the Re through the implanted optic fiber (Re stimulation condition) or delivered inside the chamber as a control (control condition). The number of seizures was counted and averaged across three sessions of Re stimulation and three sessions of control condition (Fig. 5, C to E). Optogenetic activation of Re caused a strong reduction in the seizure incidence compared to control condition over the full 1 hour of recording (Fig. 5E; control: 114 ± 7 seizures/hour; Re stimulation: 67 ± 9 seizures/hour; paired Student’s *t* test, *n* = 10, *P* = 0.0007). This 20-Hz activation of Re did not significantly alter running speed on the cylindrical treadmill (Fig. 5F; control: 10 ± 2 cm/min; Re stimulation: 25 ± 7 cm/min; Wilcoxon signed-rank test, *n* = 10, *P* = 0.1). During the 1-min-long stimulation blocks, the seizure rate was close to zero (Fig. 5G; control: 16.8 ± 1.3 seizures/10 min; Re stimulation: 1.5 ± 0.8 seizures/10 min; Wilcoxon signed-rank test, *n* = 10, *P* = 0.006). SWDs resumed after an average of 108 ± 15 s following cessation of 20-Hz Re stimulation. Together, these data show that Re stimulation can negatively modulate seizure incidence in Scna8^+/−^ mice.

It has previously been shown that optogenetic excitation or inhibition of thalamic neurons can induce SWD-related seizures (*16*, *17*), especially in the ventral posteromedial neurons. By contrast, optogenetic stimulation of Re at 7.5 Hz, the main frequency for absence seizure in Scn8a^+/−^ mice (*18*), did not result in SWDs, consistent with the idea that limbic circuits including Re have little to no involvement in SWD generation (fig. S5, A to C). Furthermore, low-frequency stimulation of Re at 1 Hz was not sufficient to reduce seizure incidence, unlike 10- and 20-Hz trains (fig. S5, D and E).

### Scn8a^+/−^ mice show deficits in cognitive flexibility

**Fig. 6.**
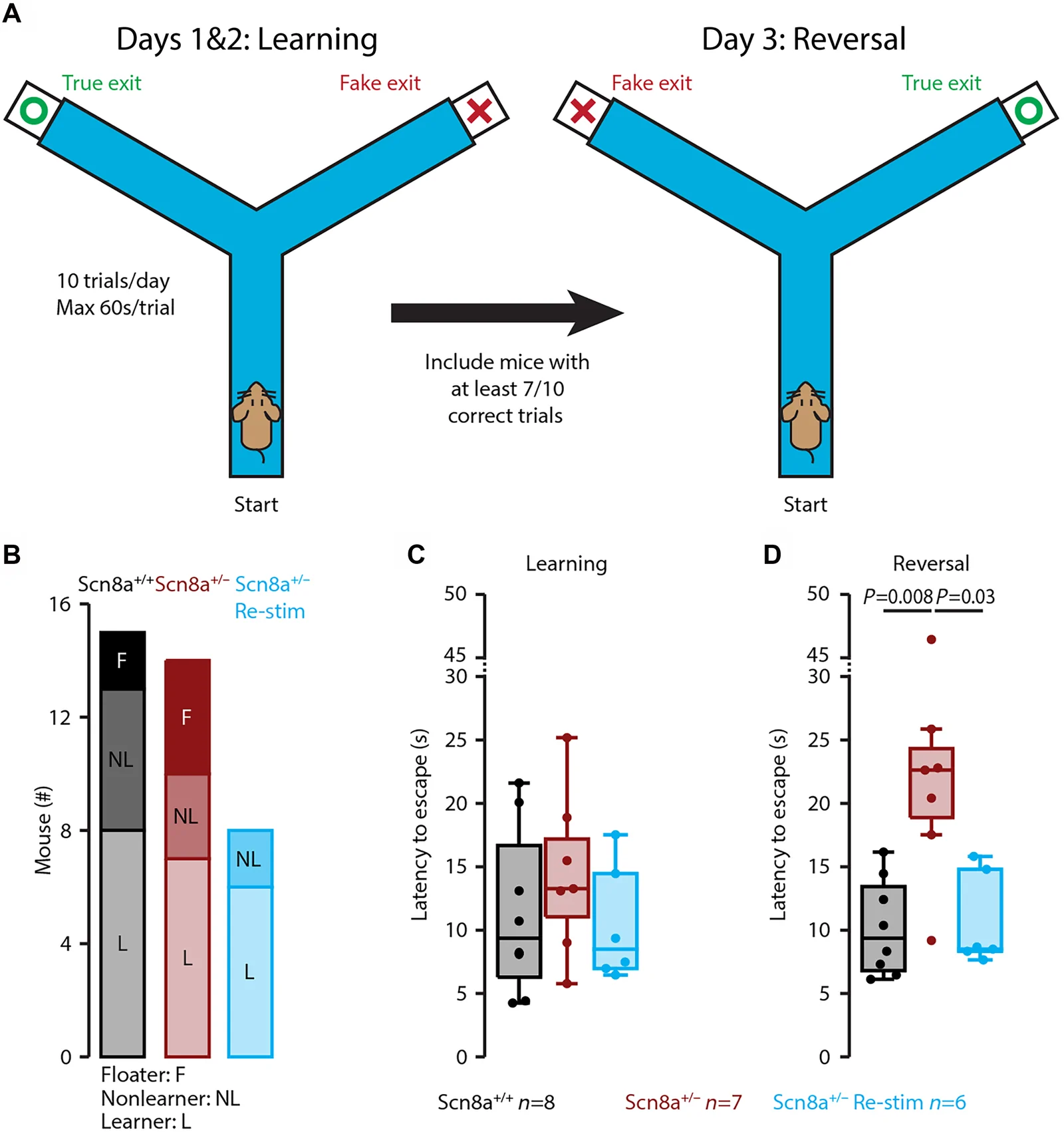
Scn8a^+/−^ mice display perseverant behavior during reversal learning in the swimming Y maze. (**A**) Schematic of the swimming Y-maze task. Mice underwent a learning phase on days 1 and 2 (left) followed by a reversal phase on day 3 (right). (**B**) Learner (L) mice reached the criterion of ≥7 of 10 correct trials on day 2 and advanced to the reversal phase. Nonlearners (NL) and floaters (F) were excluded from subsequent analyses. (**C**) Escape latency during the learning phase for Scn8a^+/+^ (black), Scn8a^+/−^ (red), and Scn8a^+/−^ with Re optogenetic stimulation (day 2). (**D**) Escape latency during the reversal phase (day 3).

Previous work showed that Scn8a^+/−^ mice display impairments in an attentional engagement task (*24*). We used a variant of the T-insert within a Morris water maze (*35*) to assess cognitive performance in Scn8a^+/−^ epileptic mice. This approach allows to measure robust indicators of learning, memory, and executive function. It requires a much shorter training period than classical water maze and does not rely heavily on exploration, motivation for novelty, or food/water deprivation. Using a Y maze with a 120° angle avoids sharp turns and makes the maze more natural for mice, reducing learning time (*36*). Thus, the swimming Y-maze variant provides a robust behavioral paradigm to detect subtle impairments in spatial learning and flexibility in Scn8a^+/−^ mice. We trained Scn8a^+/−^ and wild-type littermates in the swimming Y-maze task, where mice learned to escape through a fixed tunnel at the end of the correct arm for two consecutive days (10 trials per day; Fig. 6A, left) [adapted from (*37*)]. Mice that achieved at least 7 of 10 successful trials on the second day (escape latency <60 s without entering the wrong arm) were advanced to the reversal phase. On the third day, the escape location was switched to the opposite arm (Fig. 6A, right). Approximately half of the mice, regardless of genotype, successfully reach the learning criterion (Fig. 6B). Mice that floated passively during multiple trials without searching for the exit were excluded from analysis (two Scn8a^+/+^ and four Scn8a^+/−^ mice). Scn8a^+/−^ mice and their wild-type littermates performed similarly during initial acquisition of the task (Fig. 6C; escape latency: Scn8a^+/+^: 11.4 ± 2.3 s; Scn8a^+/−^: 14.5 ± 2.4; Student’s *t* test, *P* = 0.75). However, during reversal learning, Scn8a^+/−^ mice displayed significantly longer escape latencies (Scn8a^+/+^: 10.2 ± 1.3 s; Scn8a^+/−^: 23.6 ± 4.3; Dunn test, *P* = 0.008) compared to wild-type littermates (Fig. 6D).

To test whether enhancing Re activity could rescue this deficit, Scn8a^+/−^ mice expressing ChR2 in Re received a 20-Hz stimulation train for 1 min each day before the 10 trials of the swimming Y maze, as described in Fig. 5. This timing was chosen on the basis of the observation that seizures took an average of 108 ± 15 s to resume after the 1-min-long stimulation train, suggesting a lasting effect in the thalamocortical networks. These optogenetically stimulated mice (Scn8a^+/−^ Re-Stim) performed similarly to both nonstimulated Scn8a^+/−^ and wild-type littermates during the task acquisition (escape latency: 10.4 ± 1.8 s; one-way ANOVA: *F*_2,18_ = 0.84, *P* = 0.45; Fig. 6C). During reversal learning, optogenetically stimulated Scn8a^+/−^ mice showed escape latencies (10.7 ± 1.5 s) comparable to wild-type littermates, thereby rescuing the performance deficit observed in nonstimulated Scn8a^+/−^ mice (Kruskal-Wallis test, *P* = 0.0055; post hoc Dunn tests with Hochberg correction: Scna8^+/+^ versus Scn8a^+/−^, *P* = 0.008; Scna8^+/+^ versus Scn8a^+/−^ Re-Stim, *P* = 0.73; Scn8a^+/−^ Re-Stim versus Scn8a^+/−^, *P* = 0.026; Fig. 6D). Together, these results demonstrate that Scn8a^+/−^ mice retain intact learning capacity but exhibit impaired cognitive flexibility and that optogenetic stimulation of Re before testing rescues reversal learning performance to wild-type levels without affecting acquisition.

## DISCUSSION

Uncontrolled seizures in childhood carry devastating long-term consequences, including cognitive decline, psychiatric comorbidities, psychosocial disability, and premature mortality (*38*). Because of the strong bidirectional links between epilepsy, cognition, and psychiatric health, interventions that reduce seizures may simultaneously improve broader neurodevelopmental outcomes. Thalamic neuromodulation has already demonstrated efficacy for drug-resistant epilepsy (*39*), yet there is no consensus on the optimal stimulation target for generalized seizures, leaving a critical unmet need. The Re offers a compelling new avenue: Strategically positioned at the interface of the hippocampus and mPFC, it is integral to cognitive control and memory processes (*40*). Emerging evidence, including our findings and those of others (*22*), suggests that modulating Re activity may not only suppress seizures but also address the cognitive comorbidities that burden patients with absence epilepsy.

Previous work demonstrated how defects in mPFC parvalbumin interneurons contributed to attention deficits in this mouse model (*24*). We expand here by showing that the Scn8a^+/−^ mice also show deficits in cognitive flexibility (Fig. 6), alteration of Re recruitment of mPFC with excitation/inhibition imbalance (Figs. 2 and 3), and hypoexcitability of L1 interneurons (Fig. 4). We also show that optogenetic stimulation of Re does not trigger seizures even when applied at the seizure basic frequency (fig. S5), which contrasts with equivalent stimulation in the sensory thalamus (*32*, *33*). By contrast, Re stimulation at 20 Hz reduces seizure incidence and rescues reversal learning performance, highlighting its potential for neuromodulation.

We observed a decreased baseline firing rate of putative excitatory units in the mPFC of Scn8a^+/−^ mice compared to wild-type littermates in awake in vivo Neuropixels recordings (fig. S1A). In contrast, ex vivo recordings revealed reduced Re-mediated polysynaptic feedforward inhibition onto mPFC pyramidal neurons, while their intrinsic excitability remained unchanged (Figs. 2 and 3). At first glance, this appears paradoxical, because a decrease in feedforward inhibition is typically expected to increase principal cell activity. However, L1 interneurons can inhibit other interneuron populations, thereby disinhibiting principal cells (*41*). Consistent with this circuit architecture, we find that mPFC L1 interneurons are hypoexcitable in Scn8a^+/−^ mice, a change that would be expected to reduce disinhibition and thus contribute to the decreased baseline firing rate of putative excitatory mPFC units.

The synaptic and cellular deficits we observed ex vivo likely reflect long-term consequences of NaV1.6 loss of function and developmental compensation. In contrast, the antiseizure and procognitive effects of Re stimulation were measured acutely and therefore likely rely on transient, network-level mechanisms rather than durable cellular remodeling. Such mechanisms may include frequency-dependent thalamocortical entrainment and short-term synaptic depression that suppress pathological oscillations, effects that are inherently state-dependent and not readily captured in post hoc slice recordings. Whether chronic Re stimulation induces longer-lasting circuit adaptations remains an important question for future studies.

Our findings are limited to a single mouse model of typical absence epilepsy, the Scn8a^+/−^ line, in which a heterozygous loss-of-function mutation in the NaV1.6 channel is thought to drive hypersynchronous activity between the thalamic reticular nucleus and the sensorimotor thalamus, propagating to the cortex as SWDs (*23*). Notably, selective loss of NaV1.6 within the reticular nucleus alone is sufficient to produce absence seizures, likely through a combination of intrinsic and synaptic impairments in this structure. Beyond the direct channelopathy, Scn8a^+/−^ mice also exhibit maladaptive myelination changes secondary to absence seizures, which further promote epilepsy progression (*42*). Together, these sodium channel and myelination deficits may contribute to the altered excitability and excitation-inhibition imbalance we observed in the Re-mPFC pathway (Figs. 2 and 3). While reduced feedforward inhibition may reflect synaptic, axonal, or cellular mechanisms, we did not detect major genotype-dependent changes in inhibitory synaptic responses with direct optogenetic activation of VGAT+ neurons (Fig. 3, D to F). Rather, our findings are consistent with a contribution of interneuron hypoexcitability to diminished inhibitory output in Scn8a^+/−^ mice. In addition, impaired axonal spike propagation from NaV1.6 loss may further limit inhibitory output, although this was not directly tested here. It remains important to recognize that other genetic or pharmacological models of absence epilepsy, while converging on abnormal thalamocortical synchrony and SWDs, may not share the same circuit-level alterations seen in Scn8a^+/−^ mice. Extending these investigations across additional models will therefore be essential to distinguish model-specific features from mechanisms that are conserved across absence epilepsies.

Similarly, our study was restricted to the thalamocortical pathway linking the Re and mPFC. Other higher-order thalamic nuclei, including ventromedial (*43*), centromedial (*44*), and anterior thalamic nuclei (*45*), have also been implicated in seizure modulation. Moreover, the Re sends projections to several additional regions, such as the entorhinal cortex and hippocampus, reviewed in (*46*), which have not yet been examined for synaptic or cellular abnormalities in Scn8a^+/−^ mice. Dysfunctions within these circuits may also contribute to the cognitive impairments we observed.

The mechanisms underlying the seizure-reducing effect of Re stimulation remain to be fully defined. A knockin model of human BK-D434G channelopathy recapitulates absence seizures with behavioral arrests and shows increased early gene activation (c-Fos) and hypersynchronous bursting in ventral midline thalamic nuclei, including the Re (*22*, *47*). While the average firing rate of ventral midline thalamic nuclei was reduced during the ictal event, there was a significant increase in synchronized burst firing at the SWD positive peaks. Suppressing this midline bursting via pharmacological, optogenetic, or electrical interventions reduced seizure generation and enhanced vigilance, paralleling effects observed with physical perturbation or psychostimulants. These data suggest that midline thalamic nuclei may actively contribute to absence seizure initiation and propagation (*48*). Notably, seizure incidence is the highest during quiet wakefulness and drowsy states, whereas heightened vigilance suppresses seizures (*24*, *49*). Given the established role of the centromedial thalamus in arousal and attention (*50*), midline stimulation may act through two converging mechanisms: disrupting pathological synchronization within thalamocortical circuits and enhancing vigilance, both of which would be expected to reduce seizure incidence. Consistent with this, 20-Hz optogenetic stimulation centered on Re decreased seizure frequency in Scn8a^+/−^ mice (Fig. 6). However, we did not observe a uniform increase in locomotor activity across our cohort. This variability may reflect subtle differences in viral targeting or optic fiber placement, with possible costimulation of adjacent centromedial and ventromedial thalamus.

The mechanisms underlying the rescue of reversal learning deficits observed after Re stimulation in Scn8a^+/−^ mice remain unclear and warrant further study. Our recordings (Fig. 5) revealed that seizure reduction persisted beyond the stimulation period, indicating that trains of Re activation may exert lasting effects on thalamocortical networks. Such effects could result from a combination of acute neural mechanisms, such as depolarization block (*51*) or desynchronization of pathological oscillations as seen in Parkinson’s disease (*36*), and longer-term adaptive changes in synaptic strength (*53*). Given its connectivity with both the mPFC and hippocampus, the Re has been shown to coordinate activity in its postsynaptic targets at beta frequencies (15 to 30 Hz) during sequence memory retrieval (*54*). It is therefore plausible that stimulation of Re within this frequency range enhances coherence between the mPFC and the hippocampus, potentially serving as a neurobiological substrate for an “episodic buffer” that transiently maintains information stability in the period preceding a decision (*54*, *55*). The cognitive improvement observed in Scn8a^+/−^ mice is unlikely to result from an acute suppression of ongoing absence seizures during task performance, as these mice do not exhibit SWDs during active wakefulness (*24*), making it highly unlikely that seizures occurred while animals were performing the Y-maze task.

In summary, our findings demonstrate that the Re-targeted activation can reduce seizure incidence without exacerbating ictogenesis and rescue performance in reversal learning. By linking alterations in Re-mPFC circuitry, interneuron excitability, and cognitive flexibility deficits in *Scn8a*^+/−^ mice, we extend the role of the Re beyond cognition into seizure modulation and cognitive restoration. These results support the Re as a promising target for neuromodulation strategies in generalized epilepsies while also emphasizing the need to refine stimulation parameters and assess circuit specificity. Future work should explore whether different stimulation frequencies or approaches to selectively recruit interneuron populations can optimize both seizure control and cognitive outcomes, paving the way toward translational strategies for pediatric drug-resistant epilepsy.

## MATERIALS AND METHODS

### Experimental model and subject details

All experimental procedures were performed in accordance with the guidelines of the Institutional Animal Care and Use Committees of Stanford University (protocol no. 12363). This study used young adult (8 to 15 weeks old) Scn8a^med^ mice (C3Fe.Cg-Scn8amed/J, the Jackson Laboratory; RRID: IMSR_JAX:003798) and Scn8a^med^-VGAT-ChR2+ mice obtained from crossing Scn8a^+/−^ mice with hemizygous VGAT-ChR2-EYFP mice [B6.Cg-Tg(Slc32a1-COP4*H134R/EYFP)8Gfng/J, the Jackson Laboratory; RRID: IMSR_JAX:014548]. Mice were housed in a temperature- and humidity-controlled animal house, maintained in a 12-hour/12-hour light-dark cycle, and could access food and water ad libitum.

### Viral injections

Young adult mice (P30-60) were anesthetized with isoflurane (5% induction and 2% maintenance) and received Carprofen (10 mg/kg) subcutaneously. A volume of 0.5 μl of virus encoding the ChR2 [AAV1-CaMKIIa-ChR2(H134R)_eYFP-WPRE-HGH, 10^12^ GC, 100 nl/min, Penn Vector Core, Addgene, 26969P] was delivered in Re (from bregma, anteroposterior: −0.75 mm; mediolateral: 0 mm; dorsoventral: −4 mm). Recordings were performed at least 3 weeks after viral injection to allow sufficient ChR2 expression.

### In vitro patch-clamp recording

Acute brain slices were prepared from mice following established protocols (*26*). Coronal slices (300-μm thickness) encompassing the mPFC were obtained using a vibratome (Leica VT1200S). The targeted region spanned +1.4 to +2.0 mm anterior to bregma, and recordings were performed within the prelimbic and infralimbic subregions. To create the semi-isolated L1 flap (Fig. 3A), a ~1-mm vertical incision separating L1 from L2 was made immediately after coronal sectioning under a binocular microscope. A bipolar stimulating electrode was positioned within the L1 flap, while recordings were obtained from deep-layer neurons in the adjacent intact cortical column, 100 to 200 μm from the flap, within the prelimbic and infralimbic mPFC.

Slicing was carried out in an ice-cold, oxygenated solution containing 66 mM NaCl, 2.5 mM KCl, 1.25 mM NaH_2_PO_4_, 26 mM NaHCO_3_, 105 mM sucrose, 27 mM glucose, 1.7 mM ascorbic acid, 0.5 mM CaCl_2_, and 7 mM MgCl_2_. Slices were then incubated at 35°C for 30 min in a recovery solution, followed by at least 30 min at room temperature before recording. The recovery solution contained 131 mM NaCl, 2.5 mM KCl, 1.25 mM NaH_2_PO_4_, 26 mM NaHCO_3_, 20 mM glucose, 1.7 mM ascorbic acid, 2 mM CaCl_2_, 1.2 mM MgCl_2_, 3 mM myoinositol, and 2 mM pyruvate. During recordings, slices were perfused with artificial cerebrospinal fluid (ACSF) composed of 131 mM NaCl, 2.5 mM KCl, 1.25 mM NaH_2_PO_4_, 26 mM NaHCO_3_, 20 mM glucose, 1.7 mM ascorbic acid, 2 mM CaCl_2_, and 1.2 mM MgCl_2_. Two intracellular solutions were used depending on the experimental configuration. The cesium-based solution consisted of 127 mM Cs-gluconate, 10 mM Hepes, 2 mM Cs-BAPTA, 6 mM MgCl_2_, 10 mM phosphocreatine, 2 mM Mg-ATP, 0.4 mM Na-GTP, and 2mM QX-314-Cl (Tocris, 2313), adjusted to pH 7.3 and 290 to 305 mosm. The potassium-based solution contained 144 mM K-gluconate, 10 mM Hepes, 3 mM MgCl_2_, and 0.5 mM EGTA, with the same pH and osmolarity.

Series resistance (Rs) and input resistance (Ri) were monitored throughout the experiments using brief voltage or current pulses depending on the recording mode. Data were excluded if Rs or Ri varied by more than 20% over the course of the recording. No correction was applied for the measured −10 mV liquid junction potential. Two to four slices were recorded for each mouse.

Passive membrane properties were assessed in voltage clamp at a holding potential of −70 mV using 500-ms hyperpolarizing voltage steps (10 mV) or, alternatively, in current clamp from the resting membrane potential. Action potential characteristics were evaluated at rheobase under current-clamp conditions using 500-ms depolarizing current injections of increasing amplitude.

ChR2-expressing afferents were stimulated using blue light (1-ms duration) delivered to the full microscopic field via either epifluorescent illumination with a light-emitting diode light source (Thorlabs M450LP2, 450 nm, 13 mW, 19 mW/cm^2^) or a laser (Laserglow Technologies, 473 nm, 8 mW, 10.8 mW/cm^2^). To isolate excitatory and inhibitory synaptic currents, mPFC neurons were held in voltage clamp at −70 mV and +10 mV, respectively. E/I ratios were calculated only in cells where both EPSCs and IPSCs were recorded using identical optogenetic stimulation. For repeated stimulation, a frequency of 10 Hz was chosen because it engages short-term synaptic dynamics while remaining within a physiologically relevant range for thalamocortical transmission.

### In vitro LFP recordings

Extracellular field recordings were performed using procedures previously described (*26*). Coronal brain slices (400 μm thick) containing the mPFC were prepared from mice expressing ChR2-eYFP in the nucleus reuniens (Re) following viral injection. Slices were placed in a continuously oxygenated, humidified interface recording chamber maintained at 34°C and perfused with oxygenated ACSF at a flow rate of 2 ml/min.

LFPs were recorded using a linear 16-channel silicon probe (NeuroNexus Technologies) with a 100-μm interelectrode spacing, oriented perpendicular to the cortical laminae to span the full depth of the mPFC. Signals were amplified and digitized using a PZ5-32 preamplifier (Tucker-Davis Technologies) and processed with an RZ5D multichannel processor (Tucker-Davis Technologies). Acquisition protocols were implemented using the Real-time Processor Visual Design Studio (RPvdsEx) along with custom-written Python scripts. Data were sampled at 25 kHz and filtered between 0.1 and 500 Hz.

Optogenetic activation of ChR2-expressing axons was achieved using a 473-nm blue laser (Laserglow Technologies; 5-ms pulse duration; maximum output of 15 mW, 20 mW/cm^2^). The light was delivered in circular spots ~1 mm in diameter, targeting all cortical layers of the mPFC. Stimulation consisted of single pulses delivered every 20 s.

To dissect the synaptic contributions to the LFP, pharmacological agents were sequentially applied to the bath (Fig. 2, A to C). GABA_A_ (γ-aminobutyric acid type A) receptor-mediated inhibition was blocked with Gabazine (Abcam, ab120042; 10 μM), AMPARs with DNQX (Abcam, ab120169; 40 μM), NMDARs with APV (Sigma-Aldrich, A5282; 100 μM), and voltage-gated sodium channels with tetrodotoxin (Latoxan L8503; 0.25 μM). Each condition (baseline ACSF, Gabazine, DNQX/APV, and tetrodotoxin) was recorded for 5 to 8 min, and the final 2 min of each recording was analyzed to ensure steady-state drug effects.

To localize and quantify current flow associated with network activity, we computed the one-dimensional CSD from the LFP signals (*27*). Assuming a uniform extracellular conductivity of 0.3 S/m (*56*), the CSD was calculated by applying a second-order spatial derivative to signals from adjacent electrodes. The resulting sink and source patterns reflect transmembrane current flow within specific cortical layers. The successive pharmacological blockade of synaptic and action potential–mediated transmission allowed to disentangle the origins of the recorded sinks and sources in the mPFC.

### Chronic in vivo EEG recordings

Scn8a^+/−^ mice injected with AAV1-CaMKIIa-ChR2 into the Re were surgically implanted with an optical fiber (200-μm core diameter, 0.22 numerical aperture, 2.5-mm ceramic ferrule; Thorlabs) positioned 200 μm above the injection site. For seizure monitoring, two gold pins (1.27 mm, Mill-Max Manufacturing Corp.) were placed in contact with the surface of the primary somatosensory and motor cortices, serving as EEG electrodes. A third gold pin was implanted into the occipital bone to function as both ground and reference.

A custom metal head bar (eMachineShop) was placed and stabilized using dental cement (Metabond). Following surgery, mice were given 1 week to recover, after which they underwent 2 to 3 weeks of habituation to handling and head restraint on a cylindrical treadmill.

After a 1-hour period of quiet resting on the cylindrical treadmill, mice were recorded for 1 hour under two conditions: (i) the test condition, during which laser stimulation was delivered to the reuniens, or (ii) the control condition, during which the laser was not connected to the optic fiber implanted on the mouse head. For stimulation, blue laser pulses (473 nm; Laserglow Technologies) were delivered in 1-min trains at 20 Hz (5-ms pulse duration, 50-ms pulse interval, 3.9-mW output, 3.4 mW/cm^2^), interleaved with 5-min intervals without stimulation, and repeated throughout the recording period. Recordings were acquired using OpenEphys software (https://open-ephys.org/) at a sampling rate of 30 kHz.

Each mouse underwent three sessions in each condition (Re stimulation vs control). The seizure frequency of each session was quantified, and average seizure rates were compared between the two conditions.

### Acute in vivo Neuropixels recordings

#### Mouse preparation

Experiments were conducted on five Scn8a^+/−^ and three Scn8a^+/+^ mice that had received stereotaxic injections of AAV1-CaMKIIa-ChR2 into the Re. An optical fiber (200-μm core diameter, 0.22 numerical aperture, 2.5-mm ceramic ferrule; Thorlabs) was implanted 200 μm above the viral injection site. A stainless-steel miniature self-tapping screw (J.I. Morris Company, FF00CE125), connected to a Mill-Max pin (853-93-100-10-001000), was placed into the occipital bone over the cerebellum to serve as a reference electrode. To monitor cortical seizure activity, a 1.27-mm gold pin (Mill-Max Manufacturing Corp.) was positioned in contact with the surface of the primary somatosensory cortex to function as an EEG electrode. A custom-fabricated metal head bar (eMachineShop) was affixed to the skull and stabilized with dental cement (Metabond). Following surgery, mice were allowed to recover for 1 week and then gradually acclimated to handling and head fixation while walking on a cylindrical treadmill. Electrophysiological recordings were performed after 2 to 3 weeks of habituation.

On the recording day, a 1-mm-diameter craniotomy was performed over the right MC (anteroposterior: +1.5 to +2.5 mm; mediolateral: +1 to +2 mm) using a 0.5-mm burr (Fine Science Tools). The exposed brain surface was protected with a removable silicone elastomer (Kwik-Cast; World Precision Instruments), and animals were returned to their home cages for at least 4 hours before recordings began. During recording sessions, mice were head fixed on the treadmill, and the preimplanted optical fiber was connected to a 473-nm laser (OEM Laser Systems; 5-ms pulse duration; maximum output of 3.2 mW, 4.5 mW/cm^2^). EEG signals were recorded via the cortical gold pin and cerebellar reference screw using an RHD2132 headstage (Intan Technologies) and OpenEphys acquisition system. Immediately before probe insertion, the silicone covering the craniotomy was removed. A Neuropixels 1.0 probe, precoated with a red fluorescent dye (Vybrant DiD Cell Labeling Solution; Thermo Fisher Scientific), was mounted on a cable connected to a PXIe acquisition platform (National Instruments, NI-PXIe-1071) and carefully positioned above the cortical surface for recording.

#### Recording

The Neuropixels probe was slowly lowered into the brain at a 45° angle over a ~5-min period using a Sutter MP-285 micromanipulator. The insertion targeted a 3.8-mm span encompassing the MC and mPFC, crossing the midline. After insertion, the probe was allowed to stabilize for ~20 min before beginning data acquisition. Each recording session consisted of a 5-min baseline period, followed by 5 min of optogenetic stimulation at either 1 or 10 Hz. Stimulation trains were delivered every 10 to 20 s and included either a single pulse or two pulses (at 1 Hz), each pulse lasting 5 ms. Signals were recorded at a 30-kHz sampling rate using the OpenEphys acquisition system (https://open-ephys.org/).

#### Analysis

Spike sorting was performed automatically using Kilosort2.5 followed by manual curating using Phy2. Single-unit clusters were excluded if they exhibited interspike interval violations exceeding 0.05%, defined as the proportion of spikes occurring within the ±1.5-ms refractory window relative to the total spike count. Units that showed instability across the full 10-min recording session were also discarded from further analysis. Peristimulus raster plots and perievent time histograms were generated using custom-written MATLAB scripts (www.mathworks.com). *z*-Scores were calculated using the prestimulation baseline period (−200 to 0 ms) as the reference distribution. Single units with a spike width equal or below 0.37 ms were considered putative inhibitory neurons, while units above 0.37 ms were considered putative excitatory cells (*57*).

Absence seizures were identified visually on the basis of the characteristic EEG pattern, consisting of brief, sharp spike components followed by slower wave activity. To analyze the relationship between neuronal firing and the rhythmic seizure oscillation, the EEG signal was band-pass filtered between 6 and 8 Hz to isolate the dominant spike-and-wave frequency. The instantaneous phase of the seizure oscillation was extracted by applying a Hilbert transform to the filtered EEG signal, yielding a continuous phase angle time series ranging from 0° to 360°. Four of the five Scn8a^+/−^ mice had at least one detectable seizure during the acute recording (M0700: two seizures; M0703: three seizures; M3018: 25 seizures; M3020: 1 seizure) shown in Fig. 1. Excluding mouse M3018, the seizure rate observed during these short 10-min acute recordings for the other three mice was lower than during the longer 1-hour chronic recordings shown in Fig. 5. This difference likely reflects the intrinsic variability of absence seizure occurrence, which tends to cluster during quiet wakefulness and is reduced during periods of locomotion on the treadmill. In addition, the chronic recording configuration may be less stressful and allow animals to remain calmer overall, thereby increasing the likelihood of seizure. Each spike recorded from single units during seizures was then assigned to a corresponding phase value on the basis of its timing within this phase time series. To quantify the phase preference of neuronal firing, the seizure phase cycle was divided into 20° bins, and spike counts were computed within each bin to generate phase histograms representing firing probability across the oscillatory cycle. For each seizure event, circular statistics were used to calculate the resultant vector length and mean vector strength and then average across seizures within each mouse. The mean vector strength is a normalized version of the mean vector length reflecting phase synchrony, with values near 0 indicating uniform firing across all phases and values near 1 indicating strong phase preference. These measures were computed individually for each seizure and then averaged across seizures within each animal to obtain representative phase-locking metrics per mouse.

To assess whether optogenetic stimulation of the Re could trigger absence seizures, we quantified the temporal relationship between each light pulse and subsequent seizure initiation. Specifically, for each stimulation train, we calculated the latency to the closest following seizure by measuring the time interval between the onset of the stimulation and the start of the nearest seizure event. Seizure occurrence was then binned using 0.5-s intervals to generate a histogram of seizure probability as a function of time following stimulation. This approach allowed us to evaluate whether seizures were more likely to occur shortly after stimulation, as would be expected if stimulation of Re promoted seizure generation.

#### Histology and probe localization

At the conclusion of experiments, mice were euthanized via intraperitoneal injection of Fatal+ (pentobarbital; Vortech Pharmaceuticals), followed by transcardial perfusion with 4% paraformaldehyde in phosphate-buffered saline. Brains were postfixed overnight in 4% paraformaldehyde, rinsed in phosphate-buffered saline, and sectioned at a 100-μm thickness using a vibratome (Leica VT1000S). Sections containing the Neuropixels recording site in the mPFC and the viral injection site in the Re were mounted using 4′,6-diamidino-2-phenylindole (DAPI)–Fluoromount-G (Southern Biotech, 0100-20). Fluorescence images were acquired on a Zeiss Axio Imager M2 microscope to visualize DAPI staining, ChR2-eYFP expression, and the fluorescently labeled probe track. Neuropixels probe localization was reconstructed in three dimensions using SHARP-track (Allen CCF tools), which enables the alignment of histological sections and electrophysiological landmarks to the Allen Mouse Brain Common Coordinate Framework. On the basis of this alignment, each recording channel was assigned to its respective anatomical location within either the MC, mPFC, or OLFs. For anatomical attribution, the mPFC was defined to include the orbital, prelimbic, and infralimbic cortices; the anterior cingulate cortex; and the frontal pole, corresponding to areas 24, 25, and 32 according to the *Paxinos and Franklin's the Mouse Brain in Stereotaxic Coordinates*, Fifth Edition (*42*).

### Swimming Y-maze test

The Y maze consisted of three transparent Plexiglas arms, each 28 cm long, 8 cm wide, and 20 cm high, arranged at 120° angles. The maze was filled with water maintained at 20° to 22°C. Two of the arms were perforated and connected to black plastic pipes, with the water level adjusted to reach the base of the pipes. One pipe was occluded with a transparent barrier (false exit), while the other remained open (true exit). The escape pipe could be detached and used to transfer the mouse to a heating pad between trials. Distant visual cues were present in the room to facilitate spatial orientation: black-and-white horizontal stripes on the left wall (60 cm from the maze), a black curtain on the right, and a white rear wall.

All mice began from the same starting arm. The escape location was fixed to either the left or right arm during the first two sessions (learning phase). On the third day (reversal phase), the escape location was switched to the opposite arm. Each session included 10 trials, in which the mouse was given 60 s to find the correct escape route. Between trials, mice were transferred to a warmed recovery cage for 10 to 15 s. Scn8a^+/−^ mice expressing ChR2 in Re were optogenetically stimulated at 20 Hz for 1 min before the block of 10 trials, each day.

A trial was considered successful if the mouse escaped without entering the incorrect arm. It was marked as a failure if the mouse entered the wrong arm or failed to escape within 60 s. Mice that floated passively during multiple trials without actively searching for the exit were excluded from analysis (two Scn8a^+/+^ and four Scn8a^+/−^ mice). An additional five Scn8a^+/+^ and three Scn8a^+/−^ mice were excluded for not meeting the learning criterion (≥7 successful trials on day 2). The primary behavioral metrics analyzed were the average escape latency and the number of arm entries.

### Quantification and statistical analysis

All statistical analyses were performed using 2024 JMP 18.0.2 (JMP Statistical Discovery LLC, Cary, NC) and R programming software (3.6.1, The R Foundation for Statistical Computing, 2019). Comparisons between groups were assessed using least squares regression models with fixed effects or multivariate ANOVA. Statistical significance was determined by *F* tests on the least squares means. Post hoc comparisons were corrected for multiple testing where appropriate. Data are reported as the means ± SEM, and significance was set at *P* < 0.05. Significant exact *P* values are indicated in the main text and on figures.

## Supplementary Material

20260513-1
