# Supplementary material for: Beyond seizure control: Identifying deficits in cognitive networks in absence epilepsy
Source: Sci Adv. 2026 May 13;12(20):eaed3642. doi: 10.1126/sciadv.aed3642 (PMC13170670; doi:10.1126/sciadv.aed3642)
Supplement: Supplementary file 1 — Figs. S1 to S5 [file sciadv.aed3642_sm.pdf]

Science Advances
AAAS

Supplementary Materials for

Beyond seizure control: Identifying deficits in cognitive networks in absence epilepsy

Gil Vantomme *et al.*

Corresponding author: John R. Huguenard, John.Huguenard@stanford.edu

Sci. Adv. **12**, eaed3642 (2026)
DOI: 10.1126/sciadv.aed3642

This PDF file includes:

Figs. S1 to S5

Fig. S1.

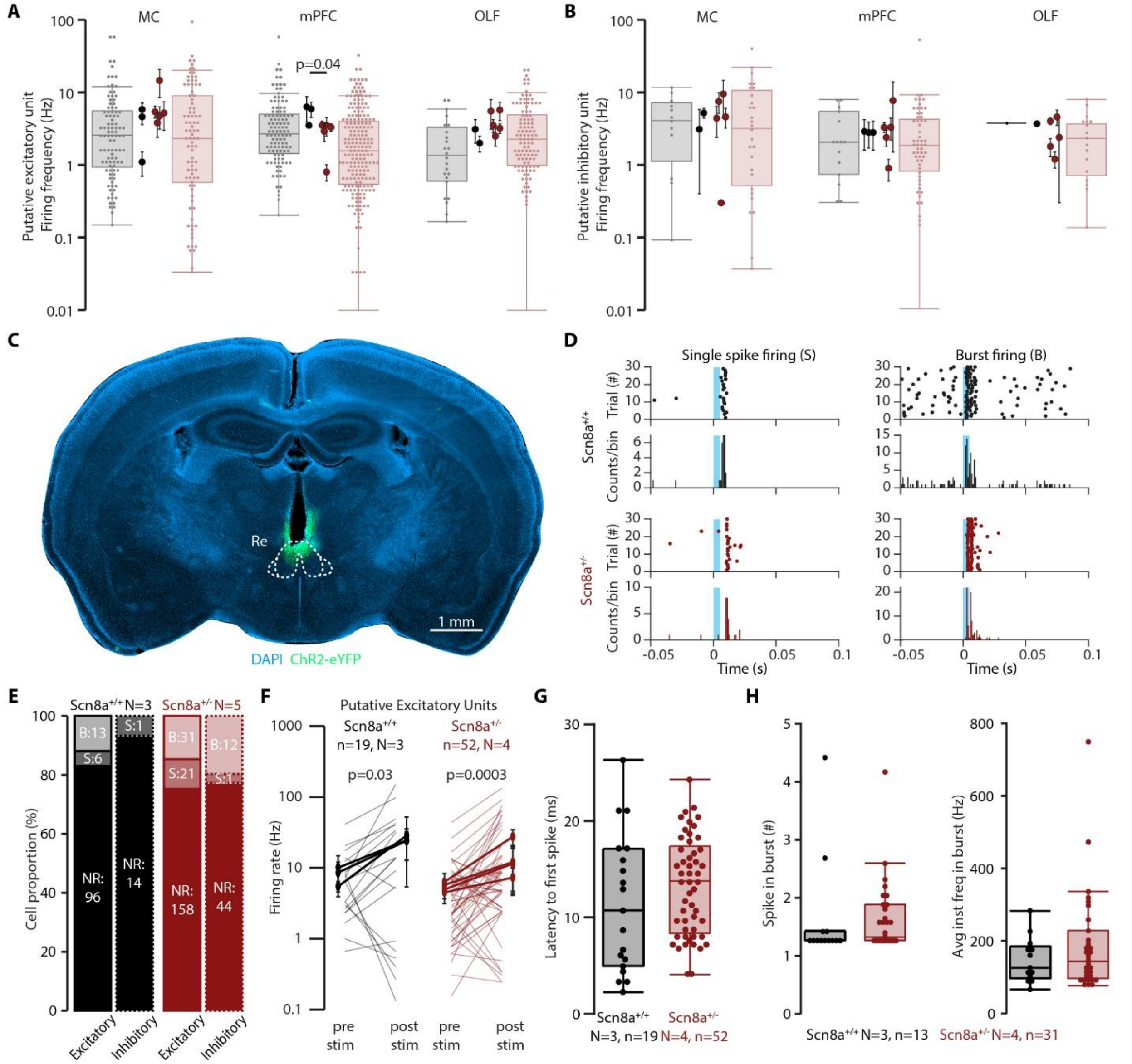

Fig. S1. Re optogenetic stimulation drives spiking of mPFC units in vivo. (**A**) Baseline firing rate of putative excitatory units in MC, mPFC and OLF from Scn8a$^{+/+}$ (black) and Scn8a$^{+/-}$ (red) mice. Semi-transparent box plots show individual units. Filled dots with error bars show mean ± sem firing rate for individual mice. (**B**) Same as (A) for putative inhibitory units. (**C**) Micrograph of a coronal brain slice with ChR2-eYFP (green) expression in Re, and DAPI-stained nuclei (blue). (**D**) Raster plots and cumulative histograms from Scn8a$^{+/+}$ (black) and Scn8a$^{+/-}$ (red) mice showing single-spike firing (left) and burst firing (right) of mPFC units following Re stimulation. (**E**) Proportion of putative excitatory and inhibitory mPFC units that did not respond (NR) or responded with a burst (B) or single spike (S). (**F**) Quantification of baseline firing frequency and post-stimulation firing frequency within 0–30 ms of laser onset for putative excitatory units in mPFC. Solid lines represent averages for each mouse. Transparent lines show individual units. (**G**) Quantification of the latency to first spike after laser onset for putative excitatory units in mPFC

(**H**) Quantification of the number of spikes per burst and the average instantaneous frequency within bursts for putative excitatory units in mPFC.

Fig. S2.

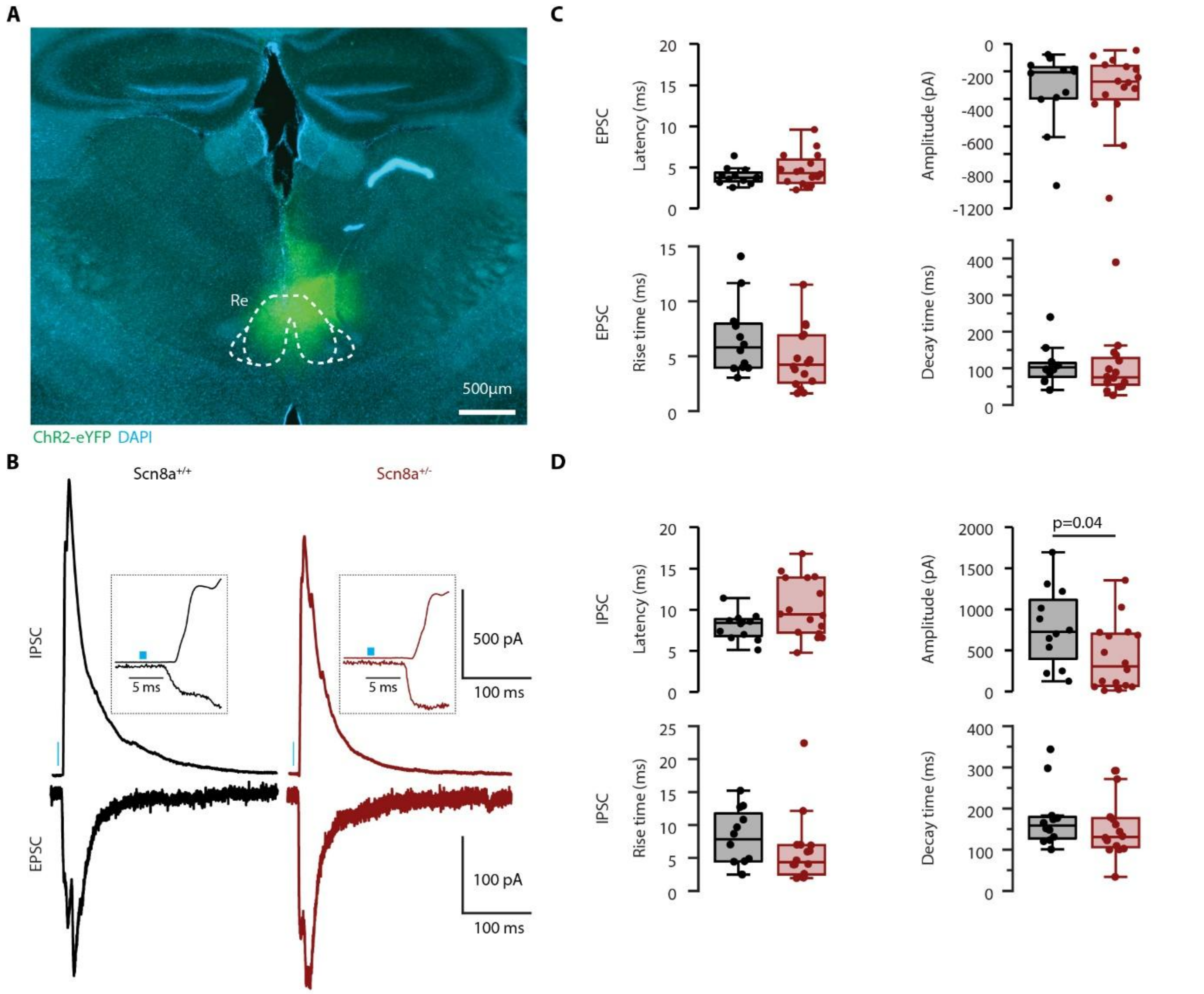

Fig. S2. Synaptic responses in L5 pyramidal neurons evoked by Re afferent stimulation. (**A**) Example micrograph of a coronal brain slice with ChR2-eYFP (green) expression in Re, and DAPI-stained nuclei (blue). (**B**) Example traces of EPSCs recorded at -70 mV and IPSCs recorded at +10 mV in the same L5 pyramidal neurons from Scn8a$^{+/+}$ (black) and Scn8a$^{+/-}$ (red) mice expressing ChR2 in Re. Inset: expanded view showing the increased latencies of IPSCs. (**C**) Quantification of EPSC properties. (**D**) Quantification of IPSC properties.

Fig. S3.

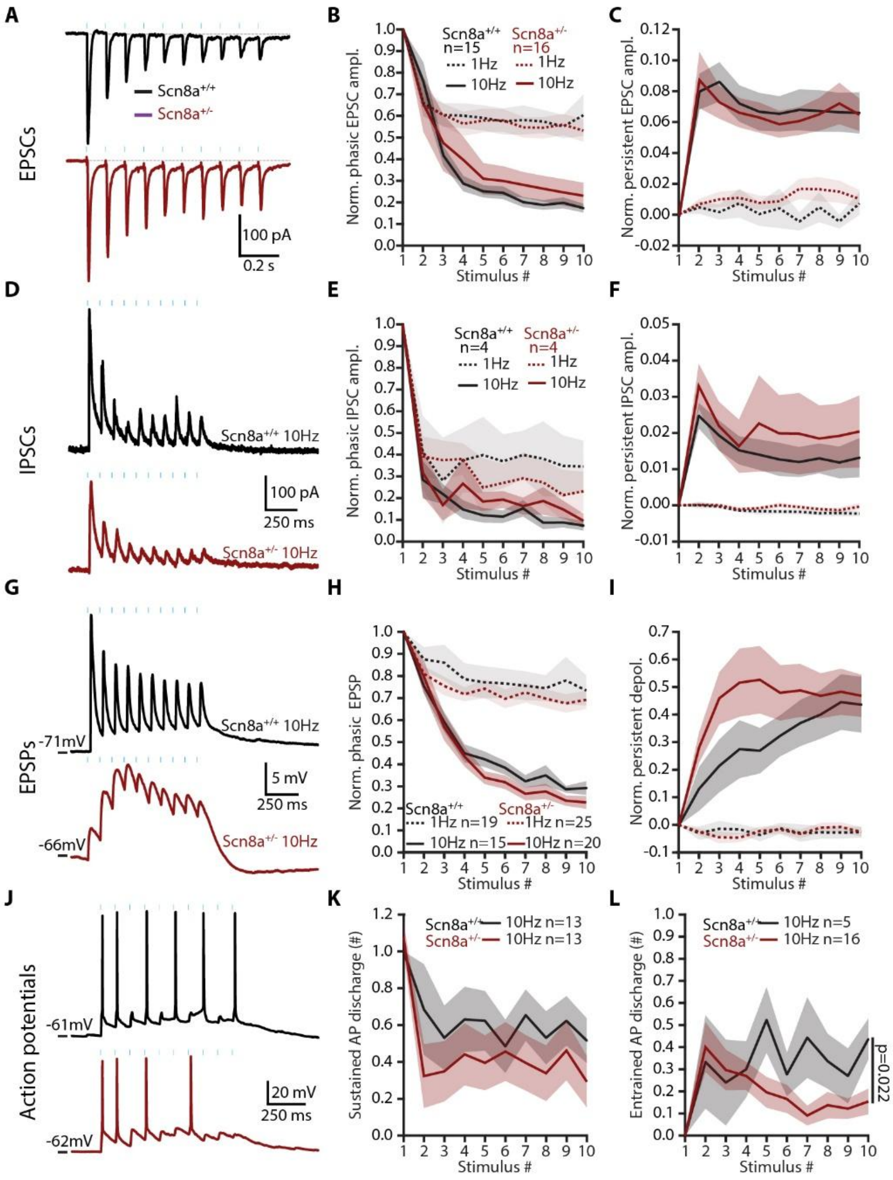

Fig. S3. Synaptic and spiking responses of L5 pyramidal neurons to repeated Re afferent stimulation. (**A**) EPSCs evoked during 10 Hz train stimulation of Re afferents. (**B**) Quantification of phasic EPSC amplitude normalized to the first EPSC. (**C**) Quantification of persistent EPSC amplitude normalized to the first EPSC. (**D–F**) Same as (A–C) for IPSCs. (**G–I**) Same as (A–C)

Hz train stimulation of Re afferents. (**K**) Quantification of sustained action potential firing. (**L**) Quantification of entrained action potential firing.

for subthreshold EPSPs. (**J**) Action potential discharge from resting membrane potential during 10
Fig. S4.

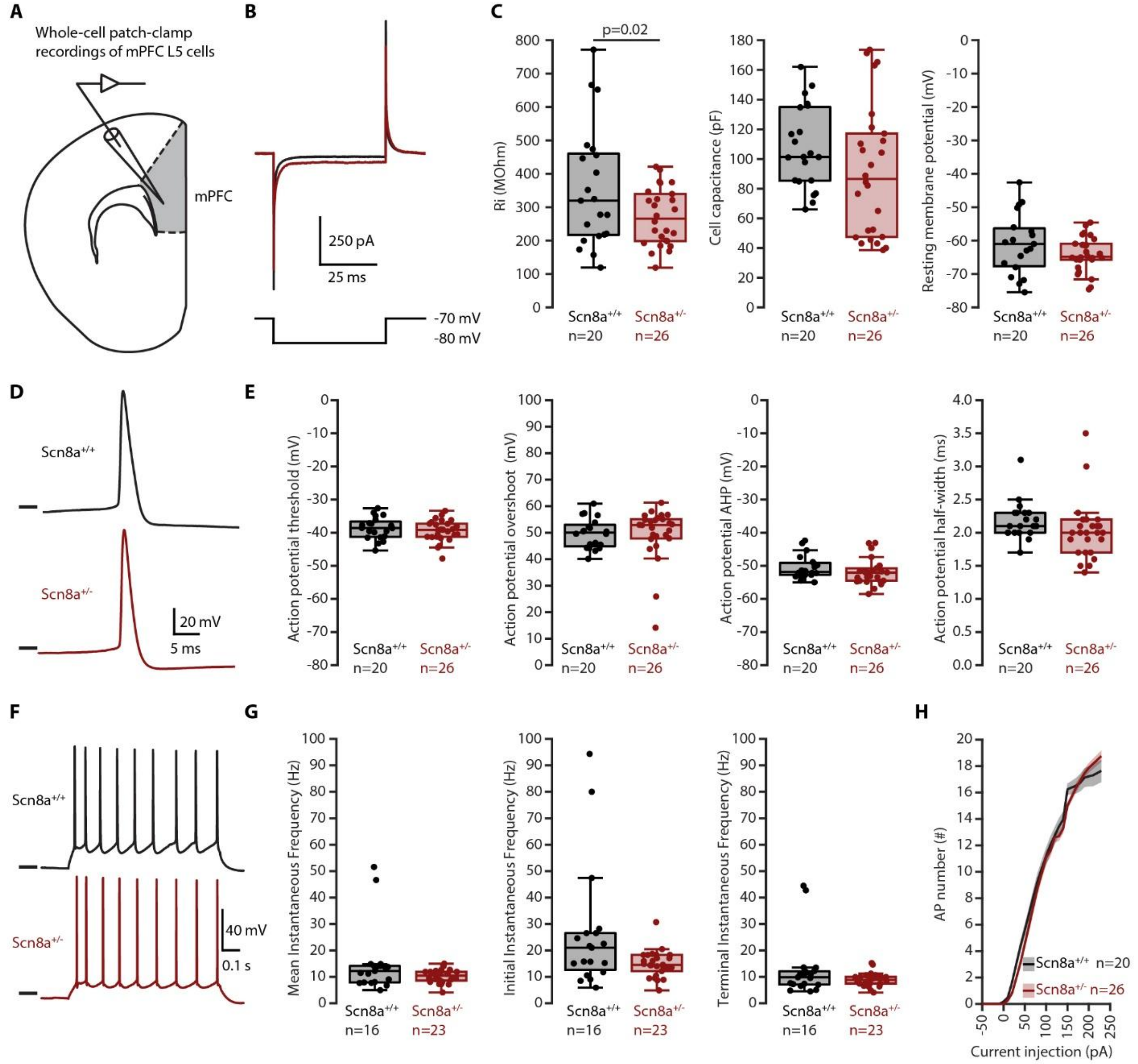

Fig. S4. Cellular properties of mPFC L5 pyramidal neurons. (**A**) Whole-cell patch-clamp recordings from L5 pyramidal neurons in mPFC slices. (**B**) Membrane response to a 10-mV hyperpolarizing step. (**C**) Quantification of input resistance (Ri), cell capacitance, and resting membrane potential. (**D**) Representative action potentials. (**E**) Quantification of action potential threshold, overshoot, afterhyperpolarization, and half-width. (**F**) Action potential discharge during positive current injection. (**G**) Quantification of mean, initial, and terminal instantaneous firing during 1-s current injections of increasing intensity.

frequency at twice the rheobase. (**H**) Quantification of the number of action potentials evoked
Fig. S5.

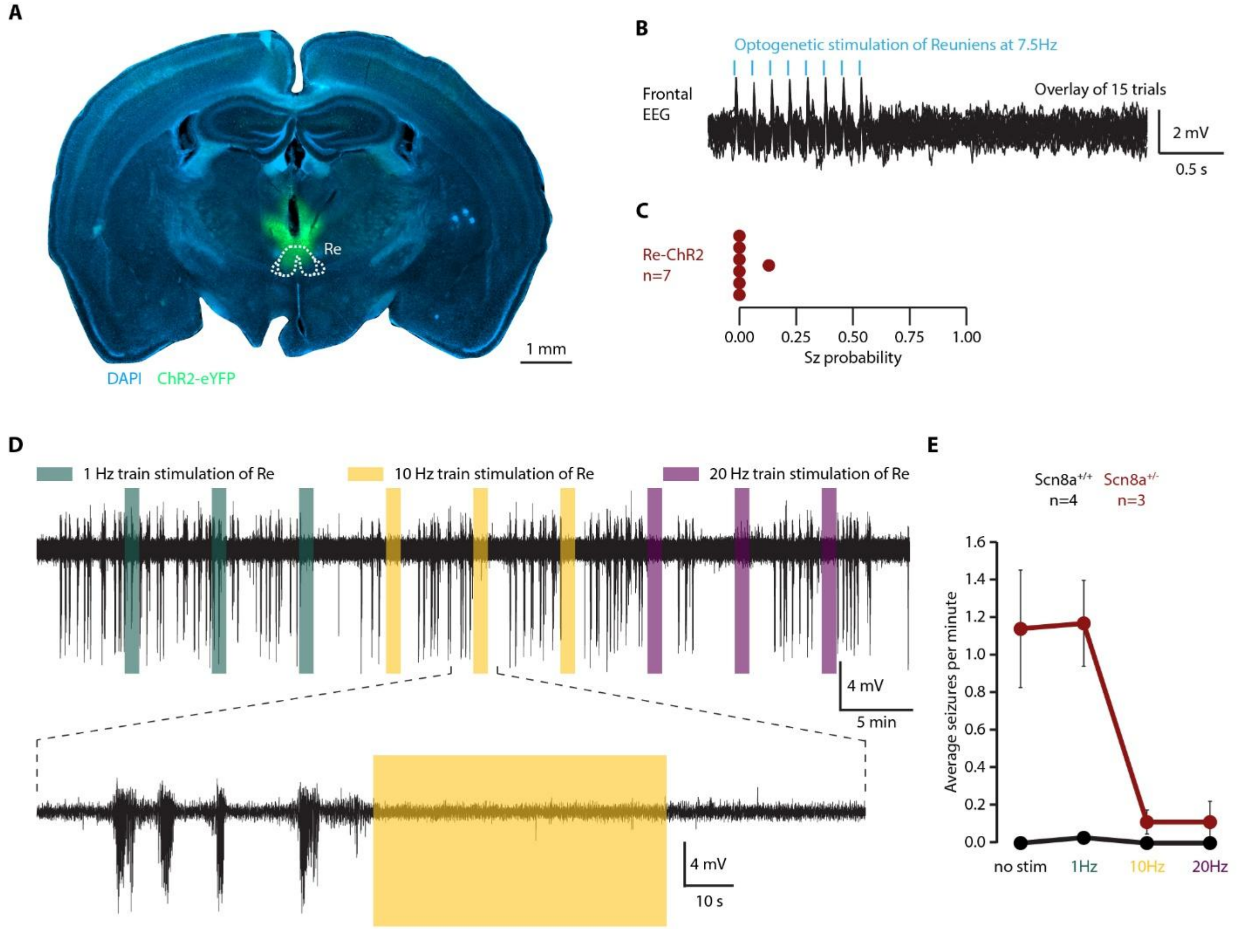

Fig. S5. Impact of the frequency of Re stimulation on SWD incidence. (**A**) Micrograph of a coronal brain slice with ChR2-eYFP (green) expression in Re, and DAPI-stained nuclei (blue). (**B**) Overlay of 15 traces showing brief deflection in the frontal EEG signal upon optogenetic stimulation of Re at 7.5 Hz. (**C**) Quantification of seizure probability for each mouse during 7.5 Hz stimulation of Re. (**D**) EEG trace from a 1 h recording. Colored bars mark periods of blue light delivery on the Re at 1 Hz (green), 10 Hz (yellow) and 20 Hz (purple). Inset: expanded view. (**E**) Quantification of the seizure rate averaged across 3 sessions for each mouse during train of stimulation at varying frequencies.
